# MACC1 regulates clathrin-mediated endocytosis and receptor recycling of transferrin receptor and EGFR in colorectal cancer

**DOI:** 10.1007/s00018-020-03734-1

**Published:** 2021-01-20

**Authors:** Francesca Imbastari, Mathias Dahlmann, Anje Sporbert, Camilla Ciolli Mattioli, Tommaso Mari, Florian Scholz, Lena Timm, Shailey Twamley, Rebekka Migotti, Wolfgang Walther, Gunnar Dittmar, Armin Rehm, Ulrike Stein

**Affiliations:** 1 Translational Oncology of Solid Tumors, Experimental and Clinical Research Center, Charité-Universitätsmedizin Berlin and Max-Delbrück-Center for Molecular Medicine Berlin in the Helmholtz-Association, Robert-Rössle-Straße 10, 13125 Berlin, Germany; 2 German Cancer Consortium (DKTK), Heidelberg, Germany; 3 Advanced Light Microscopy, Max-Delbrück-Center for Molecular Medicine, Berlin, Germany; 4 Berlin Institute for Medical Systems Biology, Max-Delbrück-Center for Molecular Medicine, Berlin, Germany; 5 Proteome Dynamics, Max-Delbrück-Center for Molecular Medicine, Berlin, Germany; 6 Tumor Immunology, Max-Delbrück-Center for Molecular Medicine, Berlin, Germany; 7 Max-Delbrück-Center for Molecular Medicine, Berlin, Germany; 8 Proteomics of Cellular Signaling, Luxembourg Institute of Health, Strassen, Luxembourg

**Keywords:** CME, Receptor recycling, MACC1, Transferrin receptor, EGFR, Colorectal cancer

## Abstract

Metastasis Associated in Colon Cancer 1 (MACC1) is a novel prognostic, predictive and causal biomarker for tumor progression and metastasis in many cancer types, including colorectal cancer. Besides its clinical value, little is known about its molecular function. Its similarity to SH3BP4, involved in regulating uptake and recycling of transmembrane receptors, suggests a role of MACC1 in endocytosis. By exploring the MACC1 interactome, we identified the clathrin-mediated endocytosis (CME)-associated proteins CLTC, DNM2 and AP-2 as MACC1 binding partners. We unveiled a MACC1-dependent routing of internalized transferrin receptor towards recycling. Elevated MACC1 expression caused also the activation and internalization of EGFR, a higher rate of receptor recycling, as well as earlier and stronger receptor activation and downstream signaling. These effects are limited by deletion of CME-related protein interaction sites in MACC1. Thus, MACC1 regulates CME and receptor recycling, causing increased growth factor-mediated downstream signaling and cell proliferation. This novel mechanism unveils potential therapeutic intervention points restricting MACC1-driven metastasis.

**Supplementary Information:**

The online version contains supplementary material available at 10.1007/s00018-020-03734-1.

## Introduction

Colorectal cancer (CRC) is the third most common cancer type in incidence worldwide, but second in mortality [1]. Although progress has been made in understanding this disease and in the availability of therapeutic interventions [2], the mortality of CRC patients remains high. Metastasis formation of CRC is the major cause of treatment failure and thus disease-related death [3]. To improve the survival of CRC patients, prognostic and causal biomarkers are needed to evaluate the risk of metastasis formation. Identifying molecular mechanisms of disease-driving factors facilitates the search for novel therapeutic intervention points.

A strong prognostic biomarker and metastasis inducer is the gene Metastasis Associated in Colon Cancer 1 (MACC1) [4]. Overexpression of MACC1 leads to progression and metastasis of many solid tumor entities, by promoting cell proliferation and migration, and thereby enhancing tumor growth and invasion [5–7]. The effect of MACC1 on promoting cancer progression and metastasis, as well as in reduced drug sensitivity has been reported in association with several cellular signaling pathways involved in carcinogenesis [7], but detailed molecular mechanisms of MACC1 in cancer progression are less well known.

The protein sequence of MACC1 is 43.7% homologous to Src homology 3 (SH3) binding protein 4 (SH3BP4) [8]. The predicted protein domains and interaction motifs of MACC1 and SH3BP4 show overlapping features: an N-terminal clathrin box, interaction motifs for the α-adaptin appendage domain of the adaptor protein 2 (AP-2) complex (Asp–Pro–Phe/DPF), the Eps15 homology domain (Asn–Pro–Phe/NPF), a central ZU5-/UPA-domain tandem, an SH3-domain and a tandem of death domains [9]. SH3BP4 has been reported to regulate the internalization of the transferrin receptor (TfR), by interacting with endocytic proteins such as clathrin and dynamin [8]. SH3BP4 localizes to TfR-containing vesicles and the perturbation of SH3BP4 expression interferes with the uptake of TfR. It has also been shown that SH3BP4 is involved in fibroblast growth factor signaling and can regulate the fate of the activated receptor, mediating its recycling or degradation, depending on the activating ligand and recruitment of SH3BP4 to the cytoplasmic tail of the receptor [10].

Considering the homology between MACC1 and SH3BP4, and the function of SH3BP4 in regulating endocytosis and receptor signaling in cancer cells, we hypothesize a role of MACC1 in clathrin-mediated endocytosis (CME) and investigated its effect on iron accumulation and receptor tyrosine kinase (RTK) signaling, and thus promoting CRC cell proliferation and metastasis.

## Materials and methods

### Shot-gun proteomics of MACC1 interactors and data analysis

For identification of the MACC1 interactome by MS (shot-gun proteomics), immunoprecipitation of SW620 cells with two polyclonal rabbit anti-human MACC1 antibodies (HPA020103, HPA020081, Sigma, St. Louis, USA, 2 µg) was performed 4 times independently. Samples were eluted from the affinity beads using denaturing buffer (6 M urea, 2 M thiourea, 20 mM HEPES, pH 8.0, Sigma). Proteins were converted to peptides in a two-step digestion using endopeptidase LysC (Wako, Japan) and trypsin (Promega, Madison, WI, USA). The peptides were desalted using Stage-Tips following a protocol by Rappsilber et al. [11]. The purified peptides were then resuspended in 3% trifluoroacetic acid/5% acetonitrile buffer (Sigma, Merck) and separated on a reversed-phase column (20 cm length, 75 μm ID, 3 μm Reprosil-C18, Dr. Maisch) with a gradient from 5 to 45% acetonitrile in 122 min. Peptides were ionized on a Proxeon ion source and directly sprayed into the mass spectrometer (Q-Exactive, Thermo Fisher). The recorded spectra were analyzed using the MaxQuant software package (Version, 1.2.2.5) [12] with fixed modifications set to carbamylation of cysteines and variable modifications set to phosphorylation of serine, threonine, and tyrosine, and methionine oxidation. The false-discovery rate was set to 0.1 on protein and peptide level. Statistical analysis of the data set was performed using the R-statistical software package [13].

Enrichment of functional categories among the identified proteins was performed using the online tool DAVID (v6.7) [14]: proteins were searched against a curated list of gene ontology terms that exclude very broad terms based on a measured specificity of each term (GO_FAT) and otherwise using standard parameters. Resulting functional categories were filtered for *p* values lower than 0.05 and Benjamini–Hochberg corrected *p* values lower than 10%.

### Cloning of MACC1-GFP deletion constructs

The plasmid RC224774L2 (Origene, Rockville, Maryland) was used to create cytomegalovirus (CMV) promoter-driven overexpression of human MACC1 (NM_182762) as a C-terminal GFP fusion protein. Parallel deletion of N-terminal CME binding sites occurred via Gibson assembly of a synthesized dsDNA fragment (Integrated DNA Technology, Leuven, Belgium) spanning 96 nt upstream of the MACC1 start codon (homologous to the vector sequence), missing the nucleotides 64–80 (clathrin box), 193–201 (NPF), 222–230 (NPF) and 295–303 (DPF), and ends at nucleotide 434 downstream of the MACC1 start codon. The vector was gapped with the restriction enzymes SfaAI and BseJI (Fast Digest, Thermo Scientific). A region around the previously described deletion of the SH3 domain of MACC1 [4] was amplified and cloned via Gibson assembly into the target vectors, gapped with the restriction enzymes XhoI and PshAI (Fast Digest, Thermo Scientific). The exact sequences of the synthesized fragment, the primers or the generated vectors are available on request.

### Cell culture

Human CRC cell lines SW480 and SW620 were obtained from American Type Culture Collection (ATCC). Cells were grown in RPMI 1640 and DMEM medium (Invitrogen, Germany), respectively, supplemented with 10% fetal calf serum (FCS; BIO&SELL, Germany) in a fully humidified incubator at 37 °C with 5% CO_2_. All cells were free of mycoplasma, confirmed with the MycoAlert mycoplasma detection kit (Lonza, US). GFP and MACC1 GFP constructs were stably transduced by lentiviral particles and selected via FACS, generating SW480/GFP, SW480/MACC1 GFP, SW480/MACC1ΔNT GFP, SW480/MACC1ΔSH3 GFP and SW480/MACC1ΔNTΔSH3 GFP cells, respectively. SW480/empty vector (e.v.), SW480/MACC1, SW620/sh control, SW620/sh MACC1, HCT116/GFP and HCT116/MACC1 GFP cells were previously established [6, 15].

### Protein extraction and WB

For total protein extraction, cells were lysed with RIPA buffer for 30 min on ice. Protein samples of equal amount were boiled for 5 min in NuPage (Invitrogen) sample buffer supplemented with 10% DTT. Samples were separated by SDS-PAGE and transferred onto nitrocellulose membranes (GE Healthcare, followed by immunoblotting with the indicated primary and corresponding secondary antibodies (see Supplementary Table 1). Chemiluminescence was visualized on Fuji medical X-ray films SuperRX (Fujifilm, Tokyo, Japan) using the WesternBright ECL kit (Advansta; Menlo Park, CA, USA). For membrane fractionation, cells were grown to sub-confluency, starved in serum-free medium for 1 h, and were treated with 50 µg/ml h-TF for 15 min or PBS, respectively. Cells were washed once with PBS and subjected to protein extraction from the plasma membrane with the Plasma Membrane Protein Extraction Kit (abcam), following the manufacturers’ instructions. Detection of TfR and Na/K-ATPase was performed via SDS-PAGE and WB.

### Immunoprecipitation

Cells were lysed in CoIP lysing buffer (20 mM Tris–HCl pH 7.5, 150 mM NaCl, 0.1% NP40, 1 mM EDTA, 1% Triton X 100, supplemented with complete protease inhibitor tablets; Roche) for 30 min on ice. Protein lysates were incubated over night with 4 µg of target specific antibodies (see Supplementary Table 1) at 4 °C. Antibody/protein complexes were captured via protein-G-coupled Sepharose beads (Invitrogen, Darmstadt, Germany) for 2 h at 4 °C. Beads were washed in CoIP lysis buffer three times before. Samples were incubated at 95 °C for 5 min and subjected to SDS-PAGE and WB.

### RNA extraction and quantitative PCR

Total RNA was isolated from cell cultures with TRIzol reagent (Invitrogen) according to the manufacturer’s instructions. Complementary DNA was synthesized from 50 ng of total RNA with random hexamers in a reaction mix (10 mM MgCl_2_, 1 × PCR Buffer II, 0.25 µM pooled dNTPs, 1 U RNAse inhibitor, 2.5 U Moloney murine leukemia virus reverse transcriptase; all from Applied Biosystems). Reaction was performed at 42 °C for 15 min, 99 °C for 5 min, and subsequent cooling at 5 °C for 5 min. mRNA expression of MACC1, TfR, SH3BP4, EGFR and glyceraldehyde-3-phosphate dehydrogenase (GAPDH) was measured by qRT-PCR using the GoTaq qPCR Master Mix (Promega, Germany) in a LightCycler 480 system (Roche Diagnostics) at the following PCR conditions: 95 °C for 2 min followed by 45 cycles of 95 °C for 7 s, 60 °C for 10 s and 72 °C for 20 s, in duplicates. The primers for MACC1, TfR, SH3BP4 and GAPDH were as follows: MACC1 forward, 5′ TTCTTTTGATTCCTCCGGTGA 3′ and reverse, 5′ ACTCTGATGGGCATGTGCTG 3′; TfR forward, 5′ GGCTACTTGGGCTATTGTAAAGG 3′ and reverse, 5′ CAGTTTCTCCGACAACTTTCTCT 3′; SH3BP4 forward, 5′ ACAACACCACCGAAATGGG 3′ and reverse, 5′ ATCATACCGCTGTCACTCAGT 3′; EGFR forward 5′ AGG CACGAGTAACAAGCTCAC 3′ and reverse, 5′ ATGAGGACATAACCAGCCACC 3′; GAPDH forward, 5′ GAAGATGGTGATGGGATTTC 3′ and reverse, 5′ GAAGGTGAAGGTCGGAGT 3′. Data analysis was performed with the LightCycler 480 Software, release 1.5.0 SP3. Target gene expression was normalized to GAPDH. All expression analyses were performed three times independently.

### Immunofluorescence

Cells starved in serum-free (SF)-MEM (Life Technologies) were treated for 8, 15, and 30 min with 50 μg/ml or saturating concentration of holo-transferrin (Sigma) as previously described [16] and for 8 min with 20 ng/ml EGF (Sigma) at 37 °C and then washed twice with PBS. Cells were fixed with 4% paraformaldehyde/PBS for 15 min at room temperature. Fixed cells were quenched for 20 min with 0.1 M glycine, washed with 0.2% Tween-20/PBS (PBS-T) and permeabilized with 0.2% Triton X-100/PBS (PBS-X) for 2 min. The coverslips were incubated 1 h in 5% albumin IgG free serum/PBS at room temperature, washed twice with PBS-T followed by incubation with primary antibodies (see Supplementary Table 2) in 2.5% albumin IgG free/PBS, overnight at 4 °C. After washing with PBS-T, coverslips were incubated with their respective secondary antibodies for 1 h at room temperature. Coverslips were incubated with DAPI (Sigma) for 3 min, and mounted with Dako Fluorescent mounting medium (Agilent). Slides were examined via a Leica SP5 fluorescence confocal microscope. Images were sampled at a resolution of 1024 × 1024 pixels, using a 63 × (NA 1.5) objective, a 5 × software zoom and a 10–15 z-step size of 0.2–0.3 μm. Quantification of colocalizations was performed using Imaris 8 (Bitplane, South Windsor, CT, USA), using the Imaris colocalization module. Representative histograms of overlapping fluorescence were determined using the Leica SP5 TCS program and expressed in arbitrary units (A.U.). Images were normalized to the background signal. Quantification of triple-localization was performed by the BlobProb plugin [17] for ImageJ [18], using a threshold of ‘5’ for all three channels and counting the number of identified colocalized blobs after triple-staining per cell, analyzing three consecutive confocal stacks per image. For compartmental analysis, images were quantified using the ImageJ software. For the receptor analysis, signals were quantified as integrated density. For the compartment-specific analysis of the receptor, masks were obtained from the EEA1–, LAMP1- and RAB11-channels, corresponding to the endosomes, lysosomes and recycling endosomes, respectively. The obtained masks were superimposed on each z-stack to the receptor channel, to obtain compartment-specific filtered images. Individual cells were then selected and circumscribed with a ROI, followed by measurement of the integrated density. For the endosomal count, images were filtered via a Gaussian blur (*σ* = 2), stacks were thresholded to binary images, overlapping particles were separated via watershed, and endosomes were detected using 0.3–6 μm^2^ and 0.5–1 circularity.

### Surface distribution, uptake, and recycling assays

Cells were grown to 80–90% confluency and starved for 1 h at 37 °C in SF-MEM (Invitrogen). Cells were washed once in ice-cold PBS, harvested, and resuspended in cold RPMI, supplemented with 0.2% bovine serum albumin (BSA) containing 25 µg/ml Alexa Fluor-647-conjugated transferrin or 2.5 μg/ml Alexa Fluor-647-conjugated EGFR (both Thermo Fisher). Cells were incubated for 1 h on ice to stain the surface located receptors. Time-resolved EGFR surface abundance was performed by binding of EGF (20 ng/ml; 1 h on ice) to the receptor, prior to internalization over the indicated time and subsequent staining of surface located EGFR. Receptor internalization was allowed by shifting the pre-stained cells to 37 °C. Fluorescence intensity of internalized TfR was determined by stopping the internalization after 10 and 20 min in ice-cold PBS. Fluorescence intensity of internalized EGFR was determined by stopping the internalization after 30 min in ice-cold PBS, with or without pre-treating the cells for 4 h with monensin (10 μM, Sigma). Cells were collected and washed twice with ice-cold PBS and fixed in 1 mM EDTA, containing 1% glutaraldehyde/PBS. Fluorescence signals of labeled EGF and Tf were analyzed at a FACS LSRFortessa (BD Bioscience). Unstained cells were used as background signal while internalized signal was calculated by virtual stripping of the surface signal. The TfR recycling assay was performed as previously described [16]. For the TfR degradation assay, cells were grown to 80–90% confluency and starved at 37 °C in SF-MEM (Invitrogen). Cells were treated for 10 and 20 min with 50 µg/ml holo-transferrin (Sigma), with or without pretreatment of bafilomycin A1 (100 nM; Sigma). Total TfR protein was detected as previously described. All experiments were performed three independent times.

### Endocytic trafficking assays

pHrodo-Red-labeled Tf and pHrodo-Red-labeled EGF (both Thermo Fisher) were used for endocytic rate assays of TfR and EGFR, respectively. Briefly, cells were grown to 80–90% confluency and starved over night at 37 °C in SF-MEM. Cells were washed twice in ice-cold Live Cell Imaging solution (LCIS, Life Technologies) and labeled Tf (50 µg/ml) or labeled EGF (5 µg/ml), diluted in Live Cell Imaging solution (LCIS, Life Technologies), were added to the plate according to manufacturer’s instructions. The plates were transferred into the IncuCyte ZOOM platform inside a humidified incubator at 37 °C/5% CO_2_. At least two images per well from at least two technical replicates were taken every 5–15 min over 3–4 h, using a 20 × objective. Images were analyzed using the IncuCyte™ Basic Software. Phase and red channel acquisition time was 2200 ms. In phase contrast, cell segmentation was achieved by applying a confluence mask. An area filter was applied to exclude objects below 50 μm^2^. Red channel background noise was subtracted with the Top-Hat method of background; integrated fluorescence signal was quantified by the software after applying a mask.

### Cell line derived xenograft mouse model, immunohistochemistry and iron staining

3.0 × 10^6^ cells of the cell lines SW480/e.v. and SW480/MACC1, respectively, were intrasplenically transplanted into 6–8-week-old SCID/beige mice with 10 animals per group. Thirty days after tumor inoculation, spleens were removed and shock frozen. Cryosections of the tumor loaded spleens were subjected to immunostaining of MACC1 (described in [4]) and Perl’s Prussian blue staining of intracellular iron. Briefly, tissue sections were allowed to thaw at room temperature, were fixed with 4% formaldehyde/PBS, washed twice with PBS and incubated for 15 min with fresh Perl’s solution (5% potassium ferrocyanide, pH 1). After 3 × wash with PBS, slides were covered with mounting medium and representative pictures were taken at a 40 × magnification (Keyence BZ-X810). Pictures of representative cell line-derived tumor sections stained for MACC1 were taken at a 10 × magnification.

### EGF-mediated signaling and cell proliferation

For signaling analysis, cells were seeded in 6-well plates for 48 h and after starvation with SF-MEM cells were stimulated with 20 ng/ml EGF in RPMI (Sigma) for the indicated time points. Cells lysates were subjected to WB and immunostaining as previously described. For determination of anchorage-dependent cell proliferation, cells were plated into 96-well plates, and grown for 72 h in the presence of 20 ng/ml EGF (Sigma) in RPMI, supplemented with 2% FCS. The proliferation rate was measured via IncuCyte ZOOM Live-cell Analysis System (Essen BioScience). Each cell proliferation experiment was performed in duplicates, for three independent times.

### In silico analysis of MACC1 and EGFR-target genes in expression microarrays of CRC patients

Publicly available expression data of CRC tumor microarrays were obtained from the functional genomics data repository Gene Expression Omnibus (http://www.ncbi.nlm.nih.gov/geo). A total of 161 expression datasets of MACC1 and EGFR-target genes were normalized to G6PDH, combined and analyzed for direct or inverse correlation. Expression data of the following cohorts were used: GDS4381 [19], GDS4513 [20], GDS4516 [21] and GDS4718 [22].

### Statistical analysis

Statistical analysis was performed using GraphPad prism software (v5.01, GraphPad software, La Jolla, CA, USA) using either unpaired two-tailed Student’s *t* tests, one-way or two-way ANOVA followed by post hoc Bonferroni correction, depending on the obtained datasets. Signal half-lives were calculated using the included function for nonlinear regression. Spearman correlation analyses were performed for gene expression data of patient cohorts. *p* values below 0.05 were considered statistically significant.

## Results

### MACC1 participates in CME by interacting with endocytic accessory and cargo proteins

**Fig. 1:**
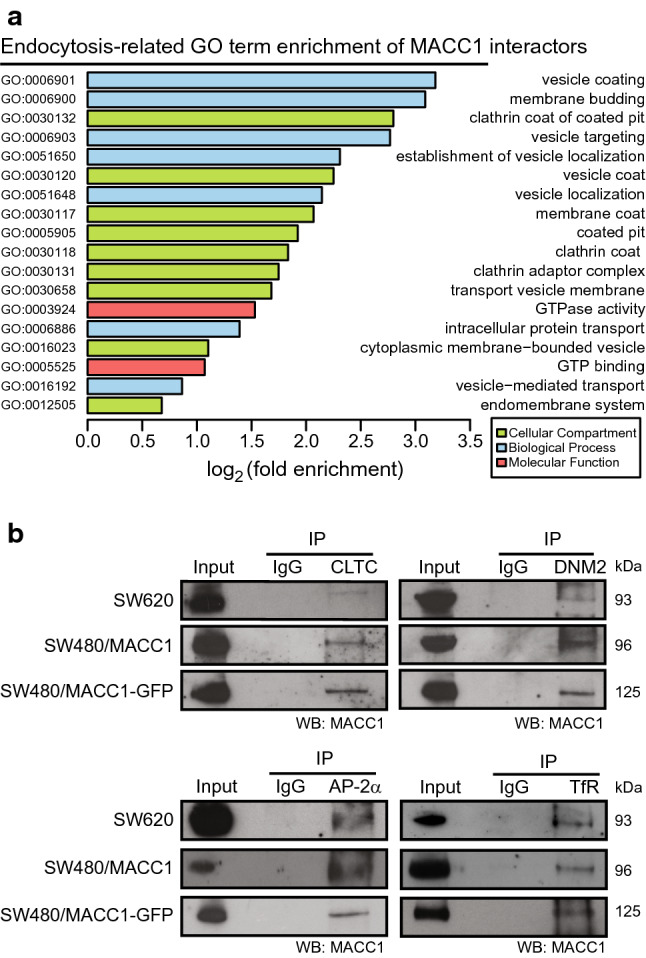
MACC1 interacts with CME factors. **a** Interaction partners of MACC1 in the CRC cell line SW620 are significantly enriched (*p* < 0.05; log_2_ > 0.5) for cellular processes. A list of enriched gene ontology (GO) terms, regarding cellular compartments, biological processes and molecular functions, includes vesicle formation and other processes of endocytosis. **b** Selected protein interactions of CME related factors with MACC1 were validated by co-IP in CRC cell lines with either high endogenous expression levels or ectopic overexpression of MACC1. *COPI* coat protein complex I, *ER* endoplasmic reticulum, *CLTC* clathrin heavy chain 1, *DNM2* dynamin 2, *AP-2α* adaptor protein 2α, *TfR* transferrin receptor 1

To address the functional impact of MACC1 during CME in CRC, we performed shotgun mass spectrometry (MS) analysis following a MACC1 pull-down in SW620 cells (see Supplementary Methods). We analyzed the MACC1-specific interactome in comparison with the reported cellular functions of SH3BP4 and found high enrichment scores of Gene Ontology terms for endocytic processes, vesicle formation and intracellular transport (*p* < 0.05; Fig. 1a, Supplementary Table 3). We identified MACC1 interactors involved in different stages of vesicle formation and trafficking, e.g., adaptor-protein (AP) complex subunits, light and heavy chains of clathrin and clathrin/AP-associated proteins, dynamin 1/2 (DNM1/2), and the CME-cargo TfR. To validate the interactome screen, we performed immuno-precipitations (IP) of MACC1 in three CRC cell models: endogenously high MACC1 expressing SW620 cells and ectopic MACC1 overexpression in endogenously low MACC1 expressing SW480 cells (SW480/MACC1 and SW480/MACC1-GFP). The interaction of MACC1 with the heavy chain 1 of clathrin (CLTC), dynamin 2 (DNM2) and the α-subunit of AP-2 (AP-2α) as endocytic accessory proteins, as well as TfR was verified by Western blots (WB) (Fig. 1b).

**Fig. 2:**
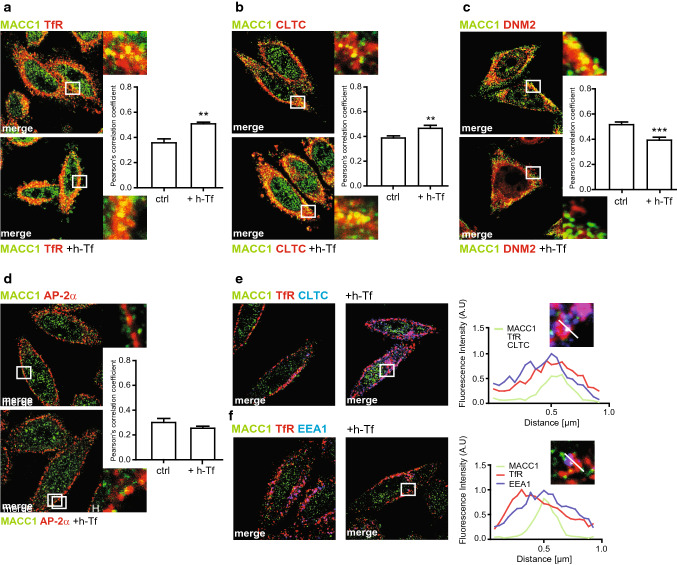
MACC1 co-localizes with TfR and CME factors after Tf-dependent TfR internalization. **a**–**d** Qualitative and quantitative assessment of co-localization of MACC1 and TfR **(a)**, CLTC **(b)**, DNM2 (**c**) and AP-2α **d** in SW480/MACC1 cells before (upper panel) and after 15 min (lower panel) stimulation of TfR internalization with h-Tf. Scale bar 10 µm. Indicated regions are displayed enlarged (× 10). Statistical analysis of Pearson’s correlation coefficients (*n* > 60 cells analyzed, *n* = 3, mean ± SEM) before and after stimulation is performed by Student’s *t* tests. e, f Qualitative and quantitative assessment of co-localization of MACC1, TfR and CLTC (**e**) or EEA1 (**f**) after triple-staining of SW480/MACC1 cells before (upper panel) and after 15 min (lower panel) stimulation of TfR internalization with h-Tf. Scale bar 10 µm. Indicated regions are displayed enlarged (× 10), and histograms show the spatial distribution of the signal intensities across indicated sections (*n* = 30 cells). All panels show merged images. Single channel images are provided in the supplement. *TfR* transferrin receptor 1, *CLTC* clathrin heavy chain 1, *DNM2* dynamin 2, *AP-2α* adaptor protein 2α, *EEA1* early endosome antigen 1, **p < 0.01, ***p < 0.001

The role of TfR in iron homeostasis allows for its use as a prognostic marker in many cancer types, including CRC [23, 24]. Cancer cells require a high iron supply to sustain their proliferation and increased TfR-dependent iron uptake is crucial in tumorigenesis [25]. When receptor internalization was triggered with holo-transferrin (h-Tf), we qualitatively and quantitatively assessed the co-localization of MACC1 with TfR, CLTC, DNM2 and AP-2α, confirming the involvement of MACC1 in TfR endocytic trafficking in support of our primary observations (Fig. 2; Figure S1).

Without h-Tf, MACC1 and TfR co-localized weakly near the plasma membrane (PM) and in cytoplasmic vesicular structures, but increased upon h-Tf mediated TfR internalization (*p* < 0.01; Fig. 2a, Figure S1a). Similarly, we analyzed the co-localization of MACC1 with CLTC, DNM2, and AP-2α during CME. MACC1 co-localized only weakly with CLTC in untreated cells, but the co-localization near the PM and in the cytoplasmic region increased 15 min after h-Tf treatment (*p* < 0.01; Fig. 2b; Figure S1b). In contrast, DNM2 and MACC1 co-localized near the PM before the induction of CME, but showed decreased co-localization after h-Tf treatment (*p* < 0.001; Fig. 2c; Figure S1c). When analyzing the distribution of AP-2α and MACC1, only a weak co-localization near the PM was noticed before h-Tf treatment, which did not change after the induction of CME (Fig. 2d; Figure S1d). These results indicate the presence of MACC1 at clathrin-coated vesicles (CCV) and the association of MACC1 to protein complexes of the ligand-stimulated endocytic machinery.

We qualitatively observed a signal overlap after triple-staining of TfR, CLTC and MACC1 in vesicular structures of SW480/MACC1 cells near the PM and the perinuclear region upon h-Tf treatment (Fig. 2e, upper row; Figure S1e). By triple-staining of MACC1, TfR and early endosomal antigen 1 (EEA1) as an endosomal marker, we observed the co-localization also of these proteins after h-Tf treatment (Fig. 2e lower row; Figure S1f). This indicates that once the CCV is formed and has pinched off the PM, MACC1 and TfR stay associated until the uncoated vesicle is fused with endosomes, suggesting additional functions of MACC1 in CME.

### Differential MACC1 expression impairs the uptake of TfR in CRC cells

**Fig. 3:**
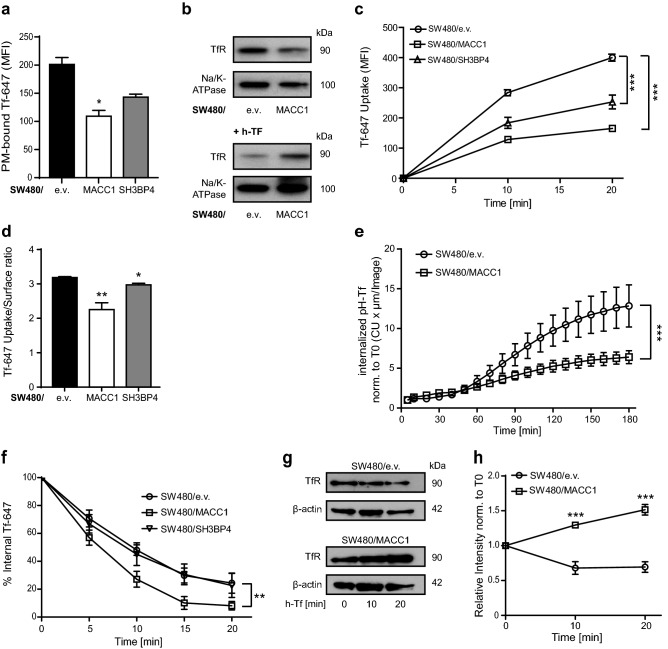
Overexpression of MACC1 modulates the surface abundance and uptake of TfR but increases its recycling. **a**, **b** Surface staining of TfR of SW480-derived cell lines, with or without h-Tf treatment. Total surface Tf-647 signal intensities (mean ± SEM) were determined by FACS (**a**). PM-bound TfR was determined by WB after membrane fractionation of untreated cells or after 15 min of h-Tf (**b**). **c** Internalized Tf-647 signal intensities (mean ± SEM) after temperature shift for subsequent time points and were determined by FACS. **d** Ratio of internalized to surface abundance signal intensities of Tf-647 (mean ± SEM). **e** Integrated pH-sensitive signal intensities (mean ± SEM) of internalized pH-Tf over time. **f** Loss of signal intensity (mean ± SEM) of internalized Tf-647 during h-Tf-stimulated CME of TfR was analyzed using non-linear regression. **g** Protein level of TfR in SW480 cells with modulated MACC1 expression before and after stimulation of TfR internalization with h-Tf at indicated time points, with β-actin as loading control. **h** Changes in TfR/β-actin ratio level upon h-Tf stimulation, after quantification of WB signal intensities and normalization to *t* = 0 min. *MFI* mean fluorescence intensity, *CU* confluency units; all experiments were performed three times independently, with ANOVA as statistical analysis (except **f**: non-linear regression), *p < 0.05, **p < 0.01, ***p < 0.001

When focusing on the surface abundance of TfR observed a significant decrease in surface-bound Alexa 647-conjugated transferrin (Tf-647) in SW480/MACC1 cells, compared to SW480/e.v. cells (*p* < 0.05, Fig. 3a), while expression levels of SH3BP4 and TfR were unaltered (Figure S2a, b). Overexpression of SH3BP4 decreased the surface signal, although not significantly, suggesting a similar but weaker role of SH3BP4 in CRC cells. We confirmed the decreased amount of TfR at the PM of SW480/MACC1 cells by membrane fractionation and WB (Fig. 3b, upper panel). After 15 min h-Tf treatment, we observed a decrease in TfR protein amounts at the PM for SW480/e.v. cells, while more TfR remained at the PM of SW480/MACC1 cells (Fig. 3b, lower panel). In addition, internalization of TfR/Tf-647 complexes over time was significantly reduced in SW480/MACC1 cells, compared to SW480/e.v. cells (*p* < 0.01, Fig. 3c). To a lesser extent, also the overexpression of SH3BP4 reduced the amount of internalized TfR/Tf-647 complexes over time. The ‘uptake to surface’ ratio of Tf-647 was lower for SW480/MACC1 and SW480/SH3BP4 cells, compared to SW480/e.v. cells (*p* < 0.05 and *p* < 0.01, respectively; Fig. 3d). As surface distribution of TfR in untreated cells and its internalization during h-Tf treatment was decreased upon overexpression of MACC1 and SH3BP4 in SW480 cells, we conclude similar cellular functions of both proteins.

We verified the role of MACC1 in TfR internalization in SW620 cells. The comparison of control cells (SW620/sh control) to MACC1-silenced cells (SW620/sh MACC1) revealed no alteration in total TfR expression (Figure S2c, d), nor any change in the surface abundance of TfR, determined by Tf-647 binding and membrane fractionation (Figure S3a, b). However, we observed a significant decrease in Tf-647 internalization 20 min after h-Tf treatment of SW620/sh MACC1 cells, compared to SW620/sh control cells (*p* < 0.05; Figure S3c, d). These data confirm a regulative role for MACC1 during TfR internalization via CME in CRC cells.

### High MACC1 expression increases recycling and impairs degradation of TfR in CRC cells

Next, we followed the accumulation of TfR in acidic cellular compartments, using Tf labeled with the pH-sensitive fluorescence dye pHrodo-Red (pH-Tf) and measured its pH-dependent signal intensity in real time. We observed significantly lower pH-Tf signal intensity in SW480/MACC1 cells 60 min after induction of CME, when compared to the control cells (*p* < 0.001; Fig. 3d). Similarly, MACC1-silencing in SW620/sh MACC1 cells increased the accumulation of TfR/pH-Tf complexes in acidic compartments, compared to SW620/sh control cells (*p* < 0.001; Figure S3e).

From endosomes, TfR/Tf complexes are mainly recycled to the PM [26], but also regulated TfR-specific degradation has been reported [27]. We assessed the recycling rate of TfR by measuring the loss of internalized Tf-647 over time. Signal intensities decreased significantly faster in SW480/MACC1 cells, compared to SW480/e.v. and SW480/SH3BP4 cells (*p* < 0.01; Fig. 3f). The signal half-life of Tf-647 in SW480/MACC1, SW480/e.v. and SW480/SH3BP4 cells was calculated as 5.4 min (95% CI 4.6–6.4; *R*^2^ = 0.95), 9.3 min (95% CI 7.8–11.4; *R*^2^ = 0.90) and 8.9 min (95% CI 7.1–11.9; *R*^2^ = 0.84), respectively.

By analyzing total protein content in SW480/e.v. and SW480/MACC1 cells, we observed increasing TfR levels in SW480/MACC1 cells after h-Tf treatment (Fig. 3g). Normalized to untreated conditions, only SW480/e.v. cells showed a reduction of TfR (to about 70%) 20 min after h-Tf treatment (*p* < 0.001; Fig. 3h).

Similarly, TfR degradation in SW620/sh MACC1 cells was triggered upon h-Tf treatment, compared to stable TfR levels in SW620/sh control cells (*p* < 0.05; Figure S3f, g). In turn, TfR degradation in SW620/sh MACC1 cells was blocked by applying bafilomycin A1, an inhibitor of the vacuolar-type H^+^-ATPase (Figure S3h, i).

**Fig. 4:**
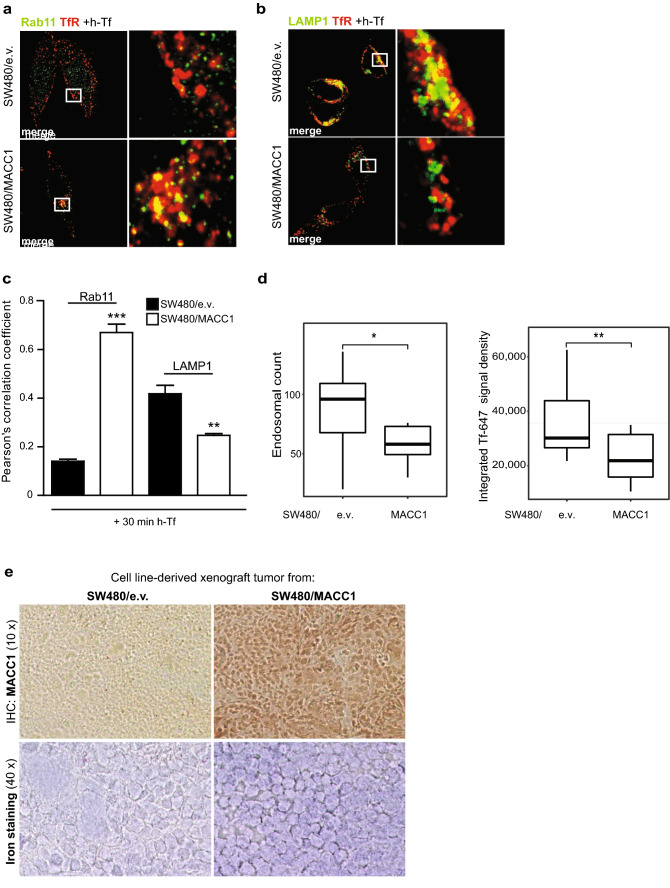
Overexpression of MACC1 localizes TfR to recycling endosomes. **a**, **b** Co-localization of TfR and Rab11 (**a**) or LAMP1 **(b**) in SW480 cells with modulated MACC1 expression after h-Tf stimulated TfR internalization. **c** Statistical analysis of Person’s correlation coefficients (*n* = 10 cells; mean ± SEM, *n* = 3) before and after h-Tf treatment are performed by Student’s *t* test. **d** Endosomal count and integration of endosome-specific Tf-647 intensity in SW480/MACC1 and SW480/e.v. cells (*n* = 20 cells; mean ± SEM). Statistical analysis was performed by Student’s *t* test. **e** Tumor sections of cell line-derived xenograft mouse models after intrasplenical transplantation. Sections of tumors derived from SW480/e.v. cells and SW480/MACC1 cells were analyzed for MACC1 expression by immunohistochemistry, and in parallel stained for intracellular iron. Scale bar 10 µm. Indicated regions are displayed enlarged (× 10). All panels show merged images. Single channel images are provided in the supplement. *Rab11* Ras-related protein Rab11, *LAMP1* lysosomal associated membrane protein 1; **p < 0.01, ***p < 0.001

Co-localization of TfR and Rab11 as a marker for recycling endosomes and the trans-Golgi network [28] clearly increased 30 min after h-Tf treatment in SW480/MACC1 cells, compared to SW480/e.v. cells (*p* < 0.001; Fig. 4a, c; Figure S4a). In contrast, co-localization of internalized TfR and the lysosomal-associated membrane glycoprotein 1 (LAMP1) in SW480/MACC1 cells decreased after h-Tf treatment, compared to SW480/e.v. cells (Fig. 4b, c; Figure S4b). The compartmental count showed increased endosomal structures in SW480/MACC1cells compared to control cells, supporting our previous observations (*p* < 0.05 Fig. 4d).

To assess, if the MACC1-dependent stabilization of TfR and its faster recycling leads to alterations of iron homeostasis, we stained tissue sections of cell line-derived tumor sections with Perls’ Prussian blue solution. Tumors derived from SW480/MACC1 cells showed substantially stronger signals when stained for MACC1, compared to tumors derived from SW480/e.v. (Fig. 4e). The higher MACC1 expression levels in the SW480/MACC1-derived tumor tissue are also associated with a stronger iron staining, compared to SW480/e.v.-derived tumors (Fig. 4e).

Thus, we conclude that elevated MACC1 expression in CRC cells caused decreased localization of internalized TfR into acidic cellular compartments, resulting in decreased receptor degradation, while reduced MACC1 expression increased its degradation. MACC1 overexpression increased the localization of TfR into recycling endosomes, which favors its recycling.

### Critical domains of MACC1 in TfR endocytosis

**Fig. 5:**
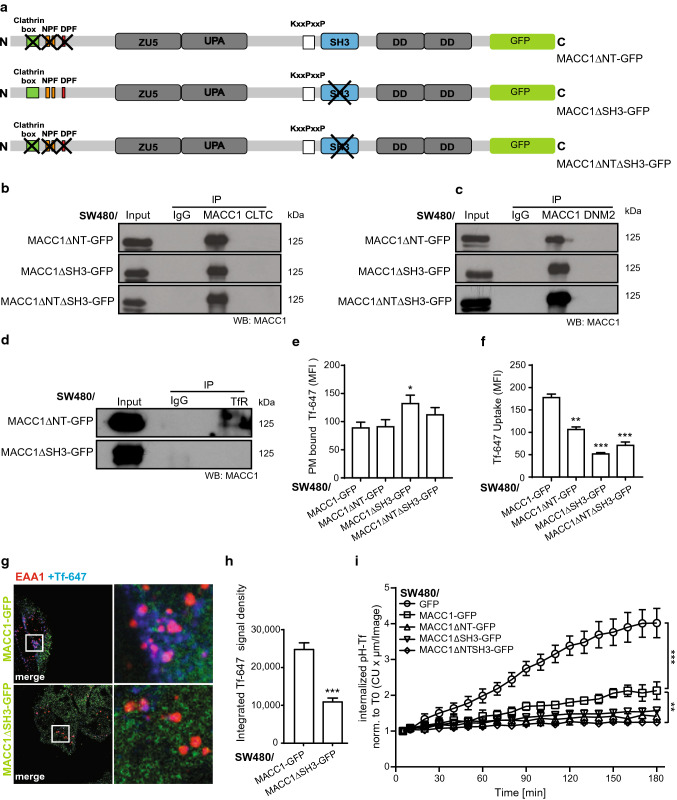
Deletion of protein interaction sites in MACC1 impacts CME-factor binding, TfR internalization and recycling. **a** Schematic view of MACC1 variants with deleted N-terminal CME-related interaction motifs (MACC1ΔNT, top), deleted SH3-domain (MACC1ΔSH3, middle), and a combination of both deletions (MACC1ΔNTΔSH3, bottom), C-terminally tagged with GFP. **b**–**d** Pull-down of MACC1 deletion variants and immunostaining for CLTC (**b**), DNM2 (**c**) or TfR (**d**). **e** Surface staining of TfR with Tf-647 of SW480-derived cell lines. Total surface Tf-647 signal intensities (mean ± SEM) were determined by FACS. **f** Internalized Tf-647 signal intensities (mean ± SEM) after temperature shift to 37 °C for 20 min. **g** Internalization of TfR/Tf-647 and co-staining of EEA1 in SW480 cells expressing GFP-tagged full length MACC1, or the SH3-domain deletion variant, after Tf-647-dependent TfR internalization. **h** Quantification and integration of Tf-647 intensity of SW480/MACC1-GFP and SW480/MACC1ΔSH3-GFP in EEA1-marked endosomal compartments. Statistical analysis of integrated Tf-647 signal densities (*n* = 10 cells; mean ± SEM) was performed by Student’s *t* test. **i** Integrated pH-sensitive signal intensities (mean ± SEM) of internalized pH-Tf over time. MFI – mean fluorescence intensity; *EEA1* early endosome antigen 1, *CU* confluency units; all experiments were performed three times independently, with ANOVA as statistical analysis (except d: Student’s *t* test), *p < 0.05, **p < 0.01, ***p < 0.001

MACC1 harbors predicted interaction sites for CME-related proteins [9]. To evaluate their importance in the MACC1-dependent alteration of the TfR cycle, we deleted the N-terminal CME-related interaction motifs (ΔNT), the SH3-domain (ΔSH3), and a combination of both (ΔNTΔSH3) (Fig. 5a). We thus generated the cell lines SW480/MACC1ΔNT-GFP, SW480/MACC1ΔSH3-GFP and SW480/MACC1ΔNTΔSH3-GFP, respectively, with SW480/MACC1-GFP as a control. We observed a re-localization of ΔSH3-MACC1 variants from the PM toward the cytoplasm, compared to full-length (fl-) MACC1 (Figure S5a). This suggests a stronger role of the SH3-domain in the MACC1-dependent effects of the TfR cycle compared to the N-terminal interaction motifs. Strikingly, the interaction of fl-MACC1 with CLTC and DNM2 was lost in any of the generated deletion variants (Fig. 5b, c), although their expression was similar to fl-MACC1 (Figure S2e, f). When we challenged the interaction of MACC1 deletion variants and TfR, protein binding still occurred in the ΔNT-MACC1 variant, but was lost upon deleting the SH3-domain (Fig. 5d). This result points to a MACC1/TfR core complex, mediated by the SH3-domain of MACC1, which can be linked to CME complexes via the N-terminal interaction motifs.

SW480/MACC1ΔNT-GFP cells showed a similar surface abundance of TfR, compared to SW480/MACC1-GFP cells. In contrast, MACC1ΔSH3-GFP and MACC1ΔNTΔSH3-GFP cells accumulated TfR at the cell surface (*p* < 0.05; Fig. 5e). Intriguingly, the uptake of TfR/Tf-647 complexes 20 min after Tf-647 treatment is progressively decreasing with the deletion of the interaction motifs, compared to fl-MACC1 (Fig. 5f).

We confirmed the impaired uptake and localization into early endosomal compartments of Tf-647 in SW480/MACC1-GFP and SW480/MACC1ΔSH3-GFP cells by confocal microscopy. We found reduced levels of internalized Tf-647 in SW480/MACC1ΔSH3-GFP cells, compared to SW480/MACC1-GFP cells (*p* < 0.001; Fig. 5g, h; Figure S4c), while compartment-specific quantification of early endosomal structures showed no alterations in SW480/MACC1ΔSH3-GFP cells, compared to SW480/MACC1-GFP cells, confirming our data.

The accumulation of TfR in late endocytic compartments is decreased in cells expressing high levels of MACC1 (as shown in Figs. 3d, S3d) and this finding is also valid for GFP-tagged full-length MACC1. Sequential deletion of interaction sites in MACC1 also impairs the pH-Tf signal intensity (*p* < 0.001; Fig. 5i).

The deletion of the N-terminal interaction motifs of MACC1 resulted in the loss of the interaction with CLTC and DNM2. Only the deletion of the SH3-domain of MACC1 strongly impaired the interaction with TfR, its internalization and endosomal localization.

### MACC1 expression promotes CME-dependent EGFR recycling

CME-mediated internalization and recycling of PM proteins can contribute to cancer progression by increasing signaling pathway intensities or altering cell adhesion [29]. With the observed role of MACC1 in the balance of TfR recycling and degradation, we analyzed its impact on CME-regulated internalization, recycling or degradation of other cancer-related signaling receptors.

**Fig. 6:**
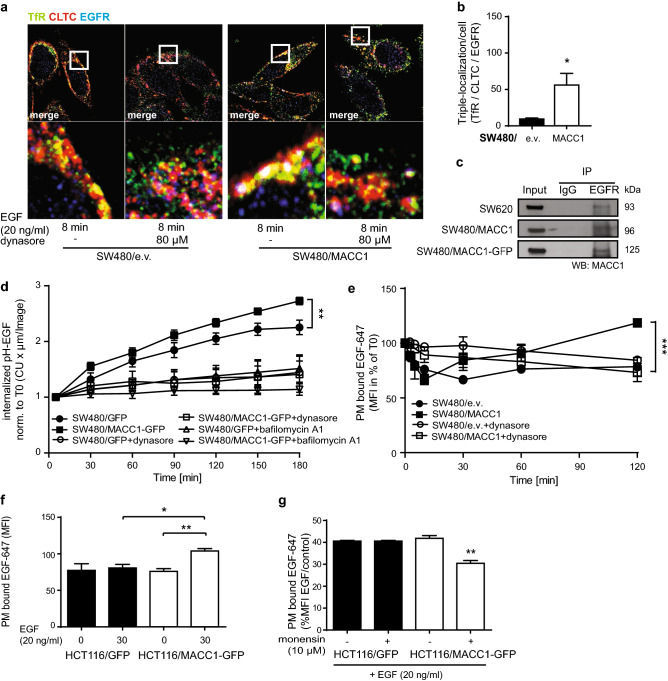
MACC1 facilitates EGFR internalization and recycling. **a** Co-localization of TfR, CLTC and EGFR after triple-staining and 8 min treatment with 20 ng/ml EGF, in SW480/e.v. and SW480/MACC1 cells. Scale bar 10 µm. Indicated regions are displayed enlarged (× 10), and histograms show the spatial distribution of the signal intensities across indicated sections (*n* = 30 cells). All panels show merged images. Single channel images are provided in the supplement. **b** Quantification of co-localization areas of TfR, CTLC and EGFR (*n* = 8; 3 confocal layers each), with statistical analysis by Student’s *t* test. **c** Co-IP of MACC1 and immunostaining for EGFR. **d** Integrated pH-sensitive signal intensities (mean ± SEM) of internalized pH-EGF over time. **e** Surface signal of EGF-647 in SW480/MACC1 and SW480/e.v. cells over time. CME of EGFR was stimulated with pre-bound EGF and blocked with dynasore. **f**, **g** Surface signal of EGF-647 in HCT116 cells with modulated MACC1 expression, before and after 30 min of EGF-stimulated EGFR internalization (**f**) or with parallel blocking of endosome acidification by monensin (**g**). *TfR* transferrin receptor 1, *CLTC* clathrin heavy chain 1, *EGF/R* epidermal growth factor/receptor, *CU* confluency units, *MFI* mean fluorescence intensity; all experiments were performed three times independently, with ANOVA as statistical analysis (except B: Student’s *t* test), *p < 0.05, **p < 0.01, ***p < 0.001

In CRC, upregulated EGFR signaling correlates with higher cell proliferation and leads to increased cell motility and survival [30]. Triggering EGFR kinase activity increases not only the surface abundance of TfR but also its co-localization in vesicles [31]. After triple-staining of EGFR, TfR and CLTC upon EGF treatment (8 min, 20 ng/ml) [32] TfR and EGFR did co-localize only weakly at the PM or in vesicles of SW480/e.v. cells (Fig. 6a; Figure S5b). In contrast, SW480/MACC1 cells showed more and larger areas of receptor co-localization after EGF treatment, also in combination with clathrin. When quantified, we found an almost six-fold increase in the co-localization of EGFR, TfR and CLTC in SW480/MACC1 cells, compared to SW480/e.v. cells (Fig. 6b). These findings confirm that TfR and EGFR follow the same early steps of CME in CRC cells and that high MACC1 levels increase the EGFR load in TfR-containing vesicles.

With EGFR and TfR sharing the same compartments, we hypothesized also an effect of MACC1 on the endocytic fate of EGFR. After ligand-stimulated CME EGFR is sorted within 30 min to either recycling endosomes or is targeted for degradation in lysosomes [32]. MACC1 is also present in EGFR-containing protein complexes, shown by co-precipitation of MACC1 after pulling-down EGFR in both SW620 and SW480/MACC1 cells (Fig. 6c). We then followed the EGFR-mediated internalization and accumulation of pHrodo-Red labeled EGF (pH-EGF) in acidic cellular compartments under differential MACC1 expression.

At low ligand concentrations, EGFR progresses further to recycling compartments. When the surrounding pH is decreasing, EGF is released and accumulates [33, 34]. At high ligand concentrations, EGFR internalization leads to lysosomal sorting and subsequent receptor degradation [32].

Treatment of SW480/MACC1-GFP cells with high concentrations of EGF (5 µg/ml) resulted in increased signal intensity over time, compared to SW480/GFP cells (Fig. 6d). When pre-treated with dynasore, a specific inhibitor of the dynamin GTPase activity, both cell lines showed equally low signals, confirming a dynamin-dependent internalization of the receptor. The increased accumulation of pH-EGF in acidic compartments upon MACC1 overexpression and high pH–EGF concentrations suggests a role for MACC1 also in the fate decision of internalized EGFR, although this effect might be masked by the activation of NCE [32].

When we analyzed the surface abundance of EGFR over time after inducing CME-driven receptor internalization with low concentrations of EGF (20 ng/ml) [32, 35] we observed a strong decrease of PM-bound EGF-647 in both cell lines within 10 min, but a significant increase of PM-bound EGF-647 in SW480/MACC1 cells from 30 min on (*p* < 0.001; Fig. 6e) while pre-treatment with dynasore inhibited CME-based receptor internalization. We validated this finding in an additional CRC cell panel with different MACC1 expression. EGF-treatment of HCT116 cells with ectopic expression of MACC1-GFP resulted in higher signal intensity after surface staining with EGF-647, compared to GFP-expressing control cells (*p* < 0.01; Fig. 6f). Additional treatment with monensin, an inhibitor of endocytic recycling [36], showed a decrease in signal activity only in HCT116/MACC1-GFP cells, but not in the control cells (*p* < 0.01; Fig. 6g). To better understand the fate of EGFR, we stimulated SW480/MACC1-GFP and SW480/MACC1ΔSH3-GFP cells with EGF-647 for 30 min and indirectly followed the routing of the EGFR/EGF-647 complex into endocytic compartments. EGF-647 was found equally co-localizing with EEA1-marked endosomal compartments in both cell lines (Figure S6a). Compartment-specific quantification showed no difference in the counts of EEA1-marked endosomal compartments (Figure S6d), as well as the integrated EGF-647 density in EEA1-marked compartments (Figure S6e). Similarly, no alterations in LAMP1-marked degradative compartments were observed (Figure S6b, d), but we recorded increased EGF-647 density in these compartments of SW480/MACC1ΔSH3-GFP cells, compared to SW480/MACC1-GFP (Figure S6b, e). In addition, although we observed no difference in the counts of Rab11-marked recycling compartments (Figure S6c, d), we recorded increased EGF-647 density in these compartments of SW480/MACC1-GFP cells, compared to SW480/ MACC1ΔSH3-GFP cells (Figure S6c, e). Taken together, this data set strongly confirms our initial hypothesis of MACC1 as a factor to indirectly enhance the routing of the EGFR/EGF-647 complex into recycling compartments, rather than into degradative compartments. Impairing MACC1 by deleting its SH3 domain results in enhanced routing of the EGFR/EGF-647 complex into degradative compartments.

Thus, the MACC1-mediated increase in TfR recycling is also observed with EGFR, increasing the availability of recycled receptors at the PM for further rounds of internalization in MACC1-high expressing CRC cells.

### MACC1 expression promotes stronger and sustained EGFR signaling

Increased recycling of activated receptors, combined with reduced degradation, leads to stronger or sustained downstream activity of the affected signaling pathways [10]. Dependent on the MACC1-promoted increase of EGFR recycling at low EGF concentrations (Fig. 6e–g), we analyzed the activation of EGFR, its internalization and its effect on downstream signaling.

**Fig. 7:**
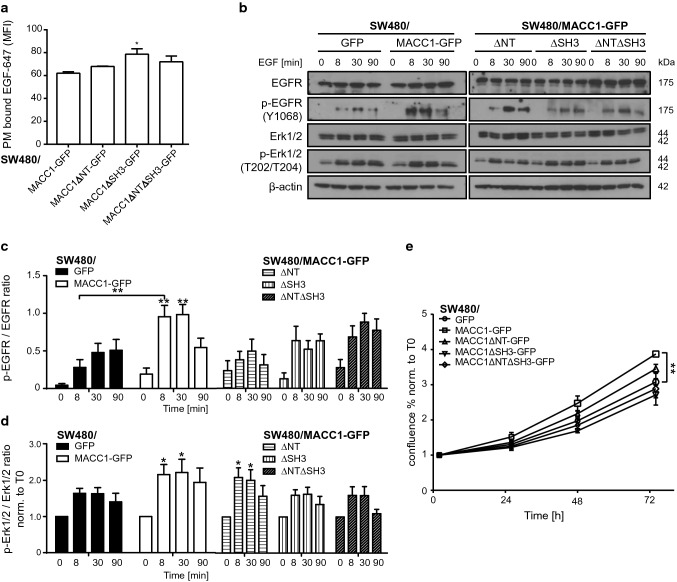
MACC1 overexpression leads to earlier and stronger EGFR Y1068-phosphorylation. **a** Surface staining of EGFR with EGF-647. Total surface EGF-647 signal intensities (mean ± SEM) were determined by FACS. **b** Protein levels of EGFR, Erk1/2, and their respective phosphorylated versions, after treatment with 20 ng/ml EGF for the indicated time points, were detected by WB with β-actin as loading control. **c**, **d** Densitometry of target specific WB staining as the ratio of phosphorylated to non-phosphorylated EGFR (**c**) and the downstream signaling proteins Erk1/2 (**d**). **e** Proliferation of SW480-derived cell lines was induced with 20 ng/ml EGF and monitored as confluency (mean ± SEM) over 72 h. *MFI* mean fluorescence intensity, *EGF/R* epidermal growth factor/receptor, *Erk1/2* extracellular signal-regulated kinase 1/2; all experiments were performed three times independently, with ANOVA as statistical analysis, *p < 0.05, **p < 0.01

We observed no change in the surface abundance of EGFR of SW480/MACC1ΔNT-GFP cells in comparison with SW480/MACC1-GFP cells (Fig. 7a). Similarly to the increased surface abundance of TfR (see Fig. 5d), we observed a significant increase in EGF-647 signal intensity of SW480/MACC1ΔSH3-GFP and SW480/MACC1ΔNTΔSH3-GFP cells (*p* < 0.05; Fig. 7a). This suggests also similar interference with receptor internalization as seen for TfR (Fig. 5e). This signal increase was not related to EGFR expression in the cell lines (Figure S2g, h).

In addition, we observed altered downstream signaling activity of stimulated EGFR (20 ng/ml EGF) by probing the phosphorylation status of the receptor and downstream signaling factors. EGFR (pY1068)-phosphorylation was peaking 30 min after EGF treatment in SW480/GFP cells (Fig. 7b, left panel). In contrast, we observed an earlier and stronger onset of maximal EGFR-phosphorylation in SW480/MACC1-GFP cells, shown by significantly higher signal intensities after stimulation (*p* < 0.01; Fig. 7c). Moreover, we observed a more than threefold increase in EGFR-phosphorylation after 8 min in SW480/MACC1-GFP cells, compared to SW480/GFP cells (*p* < 0.01; Fig. 7c). By looking at downstream signaling events, we found significantly stronger Erk1/2-phosphorylation in SW480/MACC1-GFP cells after 8 and 30 min of EGF treatment, compared to untreated cells, which was only seen to a lesser extent in SW480/GFP cells (Fig. 7d).

Cell lines expressing the MACC1 deletion variants showed intermediate EGFR-phosphorylation, indicating that the deletions inhibit, but does not abrogate the enhancing effect of MACC1 (Fig. 7c). While SW480/MACC1ΔNT-GFP cells show a significant increase of Erk1/2-phosphorylation after 8 and 30 min of EGF treatment compared to untreated cells (*p* > 0.05, Fig. 7d), this effect was lost when the SH3-domain of MACC1 was deleted.

The different pattern of EGFR activation in the panel of SW480 cells expressing MACC1 deletion variants is also seen in EGF-dependent cell proliferation. The expression of fl-MACC1-GFP significantly increased the proliferation rate (*p* < 0.01; Fig. 7e), compared to GFP expression alone. SW480/MACC1ΔNT-GFP cells grew similar to MACC1-GFP expressing cells, whereas the loss of the SH3-domain in MACC1 returns cell proliferation to the rate of SW480/GFP cells.

Taken together, overexpression of MACC1 results in earlier onset and higher intensity of EGFR-phosphorylation, leading to intensified downstream signaling via MAPK/Erk. This contributes to increased proliferation of CRC cells. The loss of N-terminal interaction motifs of MACC1 delays EGFR-phosphorylation and shortens Erk1/2. However, the deletion of the SH3-domain of MACC1 reduces its phosphorylation state and thus downstream MAPK/Erk signaling.

### MACC1 correlates with EGFR-target genes in CRC patient cohorts

Besides activating downstream signaling pathways upon ligand binding, EGFR also contributes to the expression for various oncogenes when internalized by CME [37]. As a transcriptional co-activator it regulates the expression of several target genes: cyclin D1 (CCND1), inducible nitric oxide synthase (NOS2), Myb-related protein B (MYBL2), Aurora Kinase A (AURKA), cyclooxygenase-2 (COX-2), Myc proto-oncogene protein (MYC), and breast cancer resistant protein (BCRP) (reviewed in [38]). We analyzed four publicly available expression microarray datasets from different CRC patient cohorts and observed a significant and positive correlation with five of the seven reported EGFR-target genes (Table 1, Figure S7).

**Table 1:** Correlation of MACC1 expression with EGFR-target genes in combined CRC microarray datasets

EGFR-target gene	CCND1	NOS2	MYBL2	AURKA	COX-2	MYC	BCRP
Spearman *r*	0.7739	0.7611	0.8325	0.8081	− 0.09448	0.8636	0.1076
95% Confidence interval	0.7011–0.8307	0.6848–0.8208	0.7762–0.8757	0.7447–0.8570	− 0.2499 to 0.06572	0.8167–0.8992	− 0.05254 to 0.2623
*p* value	< 0.0001	< 0.0001	< 0.0001	< 0.0001	0.2332	< 0.0001	0.1744
*p* (two-tailed)	****	****	****	****	ns	****	ns
Significant? (alpha = 0.05)	Yes	Yes	Yes	Yes	No	Yes	No
Number of pairs	161	161	161	161	161	161	161

*CCND1* cyclin D1, *NOS2* inducible nitric oxide synthase, *MYBL2* Myb-related protein B, *AURKA *aurora kinase A, *COX-2* cyclooxygenase-2, *MYC* Myc proto-oncogene protein, *BRCP* breast cancer resistant protein

## Discussion

Here we report, that MACC1, a causal and prognostic marker for CRC metastasis, is involved in the regulation of cancer-related growth factor signaling (Figure S8). By evaluating the interactome of MACC1, we found a considerable overlap in endocytosis-related GO terms to the reported function of the highly similar protein SH3BP4. Colocalization and interaction of MACC1 with the CME cargos TfR and EGFR affects their endocytic cycle by increasing receptor recycling. The increase of TfR recycling and its decreased degradation resulted in an accumulation of iron in MACC1 overexpressing cells. Similarly, increased EGFR recycling resulted in enhanced EGFR-activation and downstream signaling upon MACC1 expression, leading to increased proliferation of CRC cells.

When we validated MACC1 binding to central endocytic factors, like adaptor–protein complex, clathrin and dynamin we found the predicted linear interaction motifs (NPF, DPF) of MACC1 to be critical for the interaction. MACC1 also contains an SH3-domain, able to bind proline-rich domains (PRD) of interaction partners [39]. The deletion of this domain disrupted the interaction of MACC1 also with TfR. This type of interaction is common for recruitment and modulation of the endocytosis machinery in CCV formation [40]. Additionally, although MACC1 might not directly interact with TfR at its intracellular tail, MACC1 stays associated with TfR when it has reached early endosomal compartments, as shown by co-localization.

The ability of MACC1 to interact with CME-associated factors implies a MACC1-dependent modulation of one or several steps of CME. Focusing on receptor internalization, trafficking, degradation and recycling we employed TfR as a model system for analyzing each process. Both overexpression and knock-down of MACC1 affected the CME-dependent receptor internalization. Similar effects have been reported for the homologous protein SH3BP4, where TfR uptake was also impaired by the perturbation of SH3BP4 expression levels [8]. As MACC1 is virtually not expressed in SW480 cells, we would not consider MACC1 as a constant component of the endocytosis machinery [41], but its overexpression might interfere with CME-dependent receptor internalization due to competing protein interactions, as it has been suggested for SH3BP4 [8]. More importantly, we found a role of MACC1 in later steps of CME, when the cargo is targeted either for lysosomal degradation or recycling [26, 27]. MACC1 overexpression decreased the co-localization of TfR and LAMP1, and substantially increased the co-localization of TfR with Rab11, markers for lysosomes or recycling endosomes, respectively [42, 43]. A possible mechanism for the faster signal decrease of internalized Tf-bound fluorescence in MACC1 overexpressing cells and the reduced accumulation of Tf-647 in late endosomes is a switch from slow TfR recycling by the perinuclear or endocytic recycling compartments to rapid recycling of TfR directly by the sorting endosomes [44, 45]. On the other hand, we observe stabilized amounts of total TfR after CME stimulation in high MACC1 expressing cells. Still, it needs to be clarified whether the degradation of TfR is actively blocked, or if this effect is caused by re-routing of the entire TfR pool towards recycling.

Previous reports show a correlation between the internalization of activated EGFR and TfR [31]. We also observe a stronger co-localization of both receptors after EGF-treatment, indicating that TfR and EGFR share the same early steps of CME in CRC cells. The binding of MACC1 to TfR and EGFR, and the presence of both receptors in CCVs after the induction of CME indicates that MACC1 plays a role in the fate of EGFR. This was validated by increased recycling rates of EGFR, and a reversed effect by applying the protein transport inhibitor monensin [36]. To verify receptor internalization by CME we stimulated cells with low concentrations of EGF, which is known to cause a higher degree of subsequent receptor recycling, compared to degradation [32].

The decision for recycling over degradation is made for EFGR by the pathway of receptor internalization, depending on the ligand concentration [32]. Possible explanations for this phenomenon have already been suggested by Peschard et al*.* [46], who report a lower internalization rate and increased recycling of heterodimers of EGFR and other ErbB family members, like HER2. This is also highly likely in our cells, as elevated HER2 expression has frequently been described in CRC and confers resistance to EGFR-based therapy [47–49].

We have reported here that full-length MACC1 has an enhancing effect on EGFR-phosphorylation at Y1068. This creates an interaction site for Grb2 [50] and leads to subsequent activation of the MAPK/Erk signaling cascade. The downstream signaling cascades of EGFR, their critical protein interaction partners and cancer promoting effects have recently been reviewed [51, 52]. Although the MACC1-induced proliferation of CRC cells can now be linked to enhanced TfR and EGFR recycling, more cancer-related RTKs and respective downstream signaling pathways may be affected by this mechanism and should be the focus of future studies.

However, the intensity and duration of downstream signaling is decreased when protein interactions between MACC1 and CME-related factors are disrupted. These interaction sites have been shown to be critical for EGF-induced cell proliferation. Nevertheless, the alterations in protein complexes during CME in the presence or absence of MACC1 still need to be investigated in more detail. Potential interaction inhibitors of MACC1 and CME factors, like peptides or peptidomimetic compounds, can then be evaluated for clinical use.

## Supplementary Information

Below is the link to the electronic supplementary material.

Supplementary file1 (DOCX 5468 KB)

## Data Availability

The datasets used and/or analyzed during the current study are available from the corresponding author on reasonable request.

## Abbreviations

MACC1: Metastasis Associated in Colon Cancer 1
CME: Clathrin-mediated endocytosis
CLTC: Clathrin heavy chain 1
DNM: Dynamin
AP: Adaptor protein
EGF: Epidermal growth factor
EGFR: EGF receptor
CRC: Colorectal cancer
SH3: Src homology 3
EPS15: Epidermal growth factor receptor substrate 15
SH3BP4: SH3 binding protein 4
Tf: Transferrin
TfR: Transferrin receptor
RTK: Receptor tyrosine kinase
MS: Mass spectrometry
IP: Immunoprecipitation
PM: Plasma membrane
CCV: Clathrin-coated vesicle
EEA1: Early endosomal antigen 1
LAMP1: Lysosomal-associated membrane glycoprotein 1
Erk1/2: Mitogen-activated protein kinase ½
CCND1: Cyclin D1
NOS2: Inducible nitric oxide synthase
COX-2: Cyclooxygenase-2
MYBL2: Myb-related protein B
AURKA: Aurora kinase A
MYC: Myc proto-oncogene protein
BCRP: Breast cancer resistant protein
