# Supplementary material for: MACC1 regulates clathrin-mediated endocytosis and receptor recycling of transferrin receptor and EGFR in colorectal cancer
Source: Cell Mol Life Sci. 2021 Jan 20;78(7):3525–42. doi: 10.1007/s00018-020-03734-1 (PMC8038998; doi:10.1007/s00018-020-03734-1)
Supplement: Supplementary file 1 — Supplementary file1 (DOCX 5468 KB) [file 18_2020_3734_MOESM1_ESM.docx]

**Supplementary Methods**

qRT-PCR of target genes was performed with GoTaq® qPCR Master Mix (Promega) in a LightCycler 480 system (Roche Diagnostics) at the following PCR conditions: 95°C for 2 min followed by 45 cycles of 95°C for 7 s, 60°C for 10 s and 72°C for 20 s, in duplicates. The primers for MACC1, TfR, SH3BP4 and GAPDH were as follows: MACC1 forward, 5'‑TTCTTTTGATTCCTCCGGTGA‑3' and reverse, 5'‑ACTCTGATGGGCATGTGCTG‑3'; TfR forward, 5'‑GGCTACTTGGGCTATTGTAAAGG‑3' and reverse, 5'‑CAGTTTCTCCGACAACTTTCTCT‑3'; SH3BP4 forward, 5'‑ACAACACCACCGAAATGGG‑3' and reverse, 5'‑ATCATACCGCTGTCACTCAGT‑3'; EGFR forward 5’‑AGG CACGAGTAACAAGCTCAC‑3’ and reverse, 5’‑ATGAGGACATAACCAGCCACC‑3’; GAPDH forward, 5'‑GAAGATGGTGATGGGATTTC‑3' and reverse, 5'‑GAAGGTGAAGGTCGGAGT‑3'.

**Supplementary Table 1: Antibodies used for WB**

| **Target** | **Company and Concentrations** |  |  |
| --- | --- | --- | --- |
| **Primary Antibodies**  Anti-β-Actin (clone AC-15) | Mouse monoclonal, Pierce (1:20.000) | |  |
| Anti-MACC1 | Rabbit polyclonal, Sigma (1:1000) | |  |
| Anti-TfR (13-6800) | Mouse monoclonal, Thermo Scientific (1:1000) | |  |
| Anti-CLTC (c-20) | Goat polyclonal, Santa Cruz (1:1000) | |  |
| Anti-DNM2 (c-18) | Goat polyclonal, Santa Cruz (1:1000) | |  |
| Anti-AP2α (c-8) | Mouse monoclonal, Santa Cruz (1:1000) | |  |
| Anti-EGFR (1005)  Anti-EGFR (A-10)  Anti-p-EGFR (Tyr 1068)  Anti-p44/42  Anti- Phospho-p44/42 | Rabbit polyclonal, Santa Cruz (1:1000)  Mouse monoclonal, Santa Cruz (1:1000)  Rabbit polyclonal, Cell Signaling (1:1000)  Rabbit polyclonal, Cell Signaling (1:1000)  Rabbit polyclonal, Cell Signaling (1:1000) | |  |
| **Secondary antibodies**  Anti-rabbit-HRP (W401B) | HRP conjugated antibody, Promega (1:10.000) | |  |
| Anti-mouse-HRP | HRP conjugated antibody, Pierce (1:10.000) | |  |
| Anti-goat-HRP (sc-2020) | HRP conjugated antibody, Santa Cruz (1:10.000) | |  |

**Supplementary Table 2: Antibodies used for IF**

| **Target** | **Company and Concentrations** |
| --- | --- |
| **Primary Antibodies**  Anti-MACC1 (D-15)  Anti-MACC1 | Goat polyclonal, Santa Cruz (1:50)  Rabbit polyclonal, Sigma-Aldrich (1:100) |
| Anti-Transferrin receptor (13-6800) | Mouse monoclonal, Thermo Scientific (1:50) |
| Anti-Clathrin (X22) | Mouse monoclonal, Abcam (1:400) |
| Anti-Clathrin (CLTC) | Goat polyclonal, Sicgen antibodies (1:100) |
| Anti-Clathrin (D3C6) | Rabbit polyclonal, Cell Signaling (1:100) |
| Anti-α-Adaptin (AP6) | Mouse monoclonal, Abcam (1:100) |
| Anti-DNM2 (#3457) | Rabbit polyclonal, Abcam (1:100) |
| Anti-EGFR (1005)  Anti-EGFR (A-10) | Rabbit polyclonal, Santa Cruz (1:50)  Mouse monoclonal, Santa Cruz (1:50) |
| Endosomal markers kit (EEA1, RAB11, CLTC)  Anti-Lamp1 (D2D11) XP® | Cell Signaling (1:100)  NEB (1:100) |
| **Secondary Antibodies**  Anti-goat Alexa Fluor® 488 conjugated | Thermo Scientific (1:200) |
| Anti-mouse Alexa Fluor® 555 conjugated | Thermo Scientific (1:200) |
| Anti-rabbit Alexa Fluor® 647 conjugated | Thermo Scientific (1:200) |
|  |  |
| \| **Receptor ligands** \| \| --- \| \| Alexa-Fluor® conjugated h-Tf  Alexa-Fluor® conjugated h-EGF \| | Thermo Scientific (50 µg/ml)  Thermo Scientific (20 ng/ml) |

**Supplementary Table 3: CME-related MACC1 interactors identified by shot-gun mass spectrometry**

| **Protein Complex or Subgroup** | **Gene Name** | **Protein Name** | **Description (UniProt)** |
| --- | --- | --- | --- |
| **Clathrin** | CLTB | clathrin light chain B | major protein of the polyhedral coat of CCPs and CCVs |
|  | CLTC | clathrin heavy chain 1 |  |
| **Adaptor Protein Complex** | AP1B1 | AP1 subunit β1 | mediate both the recruitment of Clathrin to membranes and the recognition of sorting signals within the cytosolic tails of transmembrane cargo molecules |
|  | AP2A1,A2,B1,M1 | AP2 subunit α1,2,β1,µ1 |  |
|  | AP3B1 | AP3 subunit β1 |  |
| **Clathrin / AP associated** | EPS15L1 | Epidermal growth factor receptor substrate 15-like 1 | constitutive component of clathrin-coated pits that is required for receptor-mediated endocytosis. |
|  | EPN2,3 | Epsin-2,3 | role in the formation of CCVs and CME |
|  | EPN4 | Epsin-4 | role in transport via CCV from the TGN to endosomes |
|  | PICALM | Phosphatidylinositol-binding clathrin assembly protein | recruits clathrin and AP2 to cell membranes at sites of CCP and CCV assembly |
| **Vesicle fission & transport** | DNM1,2 | Dynamin-1,2 | microtubule-associated force-producing protein |
|  | DCTN1,2 | Dynactin subunit 1,2 | required for the cytoplasmic dynein-driven retrograde movement of vesicles and organelles along microtubules |
| **Ras-related Proteins** | RAB1,2,5,6,7  RAB10,11,14,35 | Ras-related GTPases | key regulators of intracellular membrane trafficking, from the formation of transport vesicles to their fusion with membranes |
| **Rab associated** | TBC1D15 | TBC1 domain family member 15 | GTPase activating protein for RAB7A |
|  | GDI1,2 | Rab GDP dissociation inhibitor alpha, beta | regulates the GDP/GTP exchange reaction of most Rab proteins |
| **Sorting nexins** | SNX1,2,6,9 | sorting nexin-1,2,6,9 | involved in several stages of intracellular trafficking |
| **Vesicle trafficking** | RME8 | required for receptor-mediated endocytosis 8 | involved in membrane trafficking through early endosomes |
|  | VPS35 | vesicle protein sorting 35 | Acts as component of the retromer cargo-selective complex (CSC). |
|  | SEC22B | SEC22 vesicle-trafficking protein homolog B | SNARE involved in targeting and fusion of ER-derived transport vesicles with the Golgi complex as well as Golgi-derived retrograde transport vesicles with the ER. |
|  | GGA1 | Golgi associated, gamma adaptin ear containing, ARF binding protein 1 | plays a role in protein sorting and trafficking between the TGN and endosomes |
|  | TMED2,10 | transmembrane-trafficking protein | involved in vesicular protein trafficking |
|  | EXOC6B | exocyst complex component 6B | involved in the docking of exocytic vesicles with fusion sites on the plasma membrane |

**Supplementary Figures and Legends**


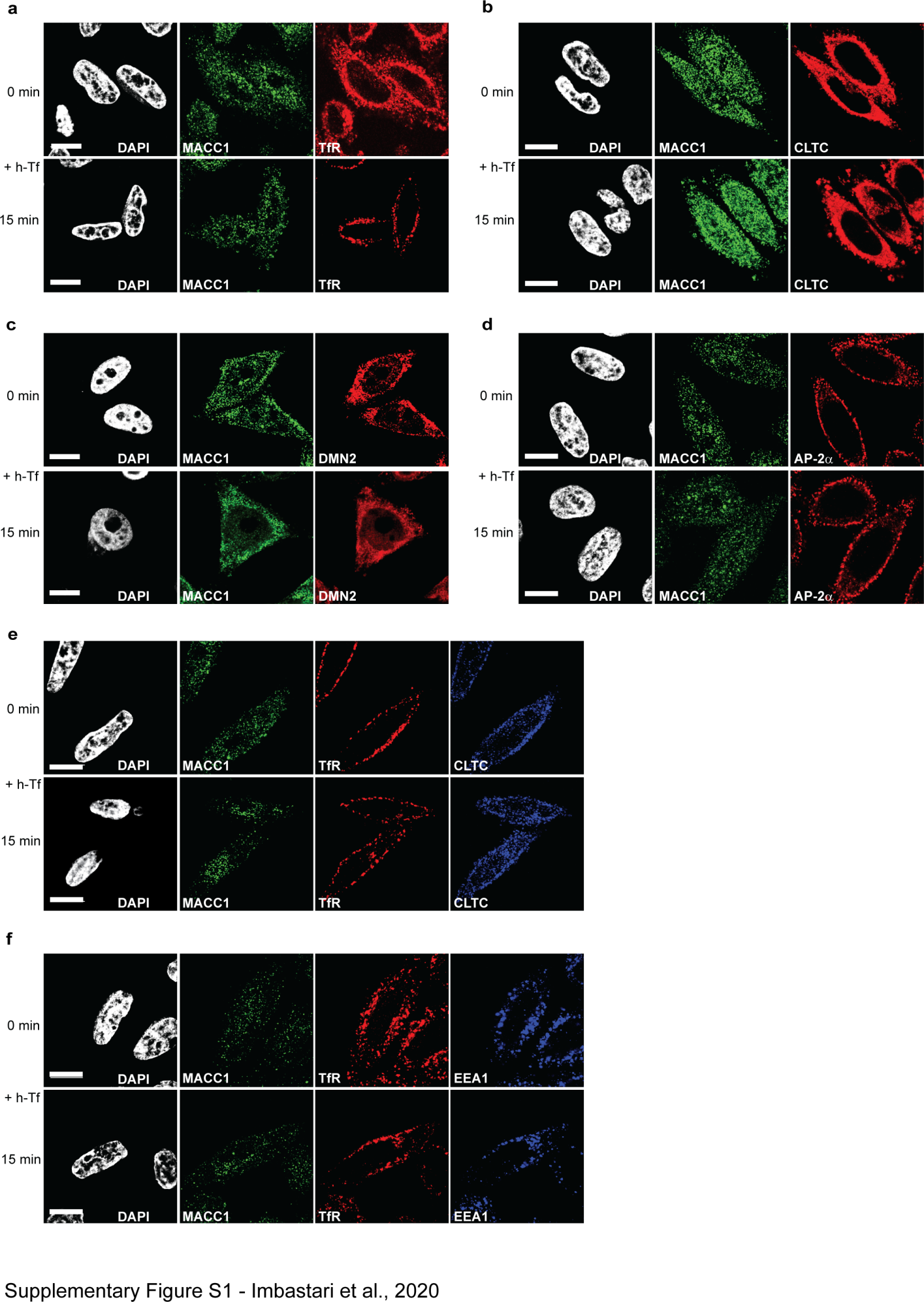


**Figure S1: Single channel images of the co-localization of MACC1 with TfR and CME factors**

**(a-d)** Qualitative assessment of the co-localization of MACC1 and TfR **(a)**, CLTC **(b)**, DNM2 **(c)** and AP‑2α **(d)** in SW480/MACC1 cells before (upper panel) and after 15 min (lower panel) stimulation of TfR internalization with h‑Tf. Nuclei were stained with DAPI. Scale bar = 10 µm. **(e,f)** Qualitative assessment of the co-localization of MACC1, TfR, CLTC **(e)** or EEA1 **(f)** after triple-staining of SW480/MACC1 cells before (upper panel) and after 15 min (lower panel) stimulation of TfR internalization with h‑Tf. Nuclei were stained with DAPI. Scale bar = 10 µm.

TfR – transferrin receptor 1; CLTC – clathrin heavy chain 1; DNM2 – dynamin 2; AP‑2α – adaptor protein 2α; EEA1 –early endosome antigen 1


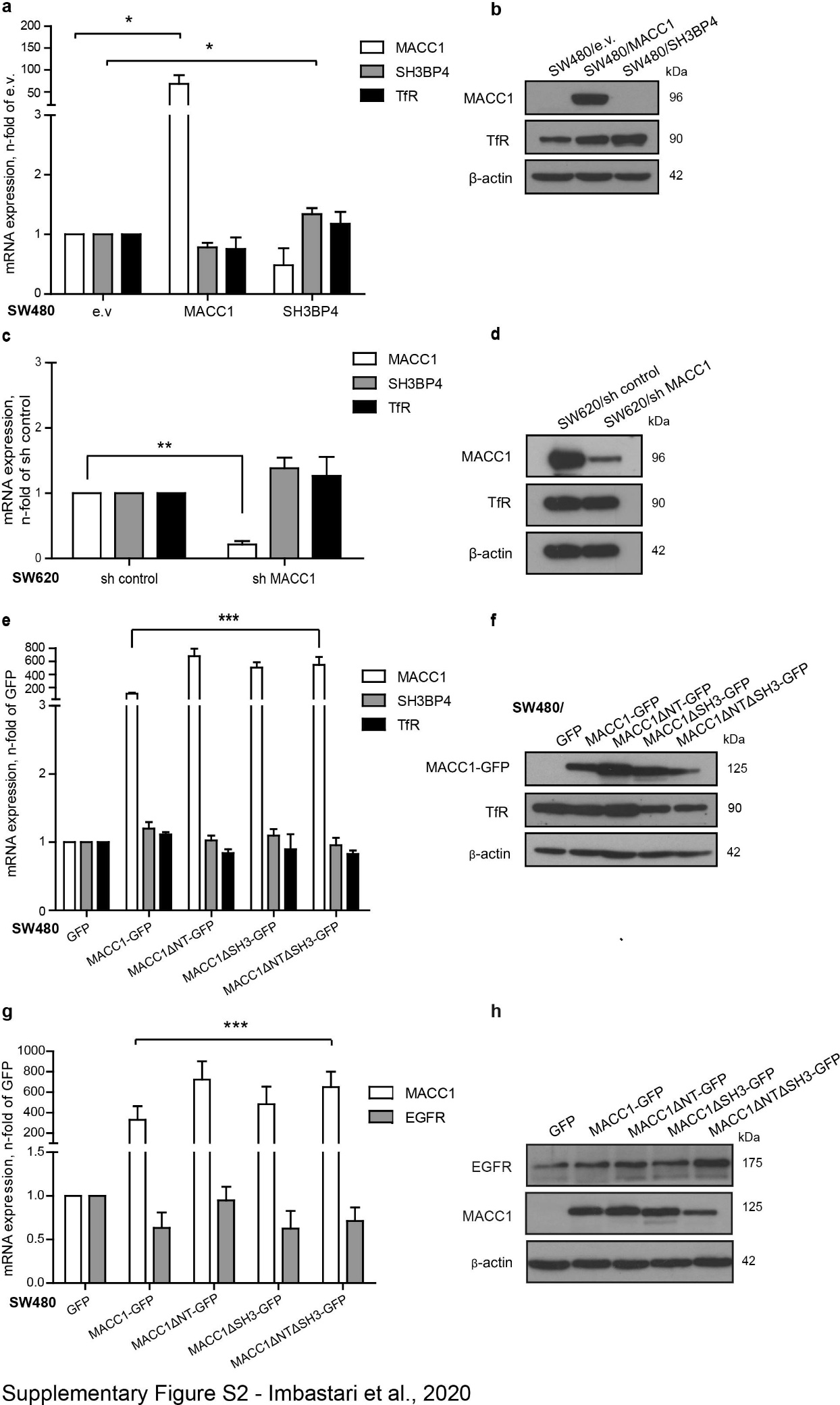


**Figure S2: Comparison of gene expression in CRC cell line models**

**(a,b)** mRNA **(a)** and protein **(b)** expression levels of MACC1, SH3BP4 and TfR in the generated CRC cell lines SW480/e.v., SW480/MACC1 and SW480/SH3BP4, respectively. **(c,d)** mRNA **(c)** and protein **(d)** expression levels of MACC1, SH3BP4 and TfR in the generated CRC cell lines SW620/sh control and SW620/sh MACC1, respectively. Loading of equal protein amounts was controlled by β actin. **(e,f)** mRNA **(e)** and protein **(f)** expression levels of MACC1, SH3BP4 and TfR in the generated CRC cell lines SW480/GFP, SW480/MACC1‑GFP, SW480/MACC1ΔNT‑GFP, SW480/MACC1ΔSH3‑GFP and SW480/MACC1ΔNTΔSH3‑G1FP cells, respectively. Loading of equal protein amounts was controlled by β‑actin. **(g,h)** mRNA **(g)** and protein **(h)** expression levels of EGFR and MACC1 in the generated CRC cell lines SW480/GFP, SW480/MACC1‑GFP, SW480/MACC1ΔNT‑GFP, SW480/MACC1ΔSH3‑GFP and SW480/MACC1ΔNTΔSH3‑GFP cells, respectively. Loading of equal protein amounts was controlled by β‑actin. All experiments were performed three times independently, with ANOVA as statistical analysis, * p < 0.05, ** p < 0.01, *** p < 0.001


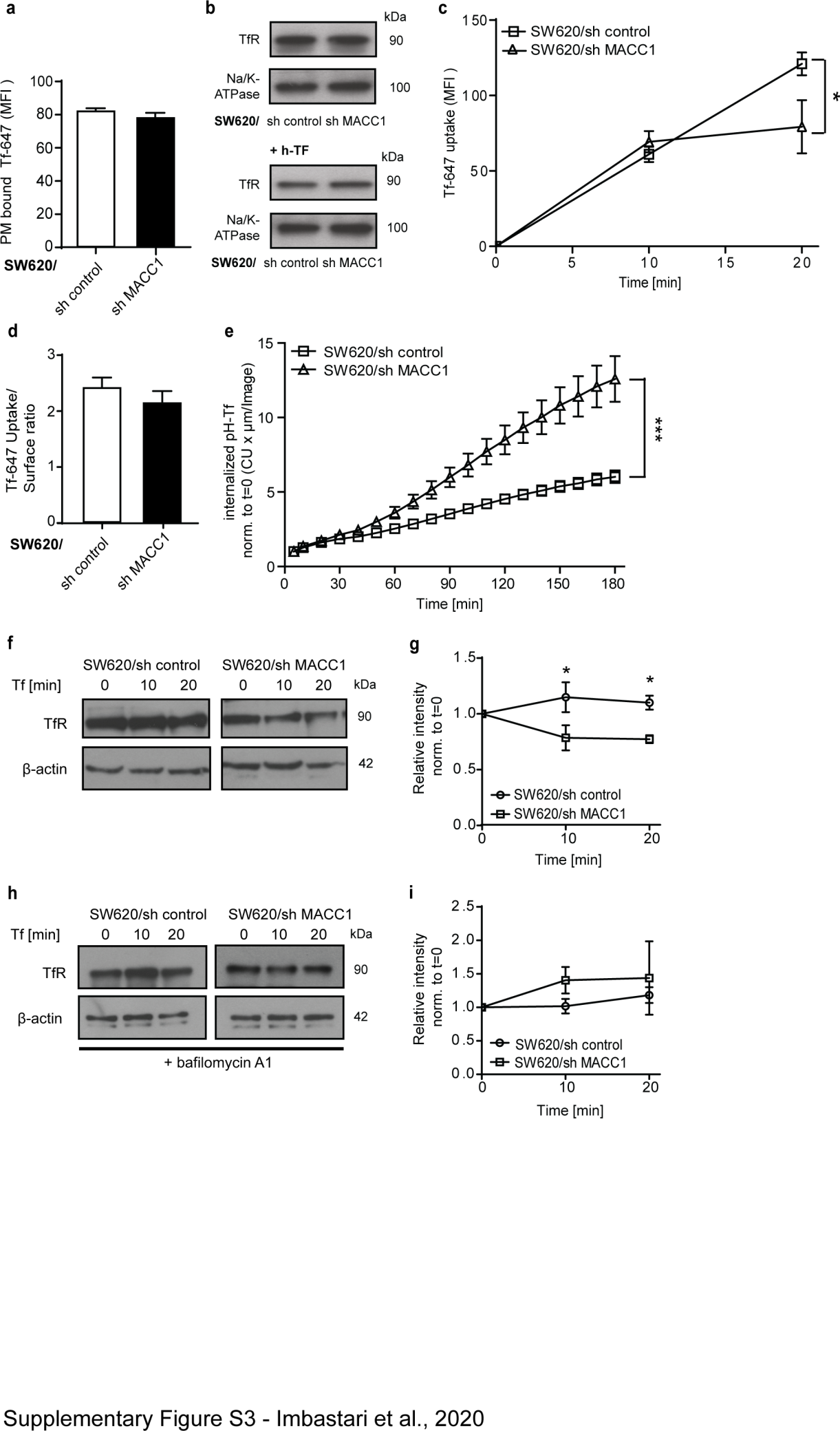


**Figure S3: Knock-down of MACC1 modulates TfR uptake, but increases its degradation**

**(a,b)** Surface staining of TfR with Tf‑647 of SW620-derived cell lines, pre and post h-TF treatment. Total surface Tf‑647 signal intensities (mean ± SEM) were determined by FACS **(a)** and WB after membrane fractionation **(b)**. **(c)** Internalized Tf‑647 signal intensities (mean ± SEM) after temperature shift for subsequent time points were determined by FACS. **(d)** Ratio of internalized to surface abundance signal intensities of Tf‑647 (mean ± SEM). **(e)** Integrated pH-sensitive signal intensities (mean ± SEM) of internalized pH‑Tf over time. **(f)** Protein level of TfR in SW480 cells with modulated MACC1 expression before and after stimulation of TfR internalization with h‑Tf at indicated time points, with β‑actin as loading control. **(g)** Changes in TfR/β actin ratio level upon h‑Tf stimulation, after quantification of WB signal intensities and normalization to t = 0 min. **(h)** Protein level of TfR in SW480 cells with modulated MACC1 expression, pre-treated with bafilomycin A1, before and after stimulation of TfR internalization with h‑Tf at indicated time points, with β actin as loading control. **(i)** Changes in TfR/β‑actin ratio level in cells pre-treated with bafilomycin A1, upon h‑Tf stimulation and after quantification of WB signal intensities and normalization to t = 0 min.

MFI – mean fluorescence intensity; CU – confluency units; all experiments were performed three times independently, with ANOVA as statistical analysis, * p < 0.05, ** p < 0.01, *** p < 0.001


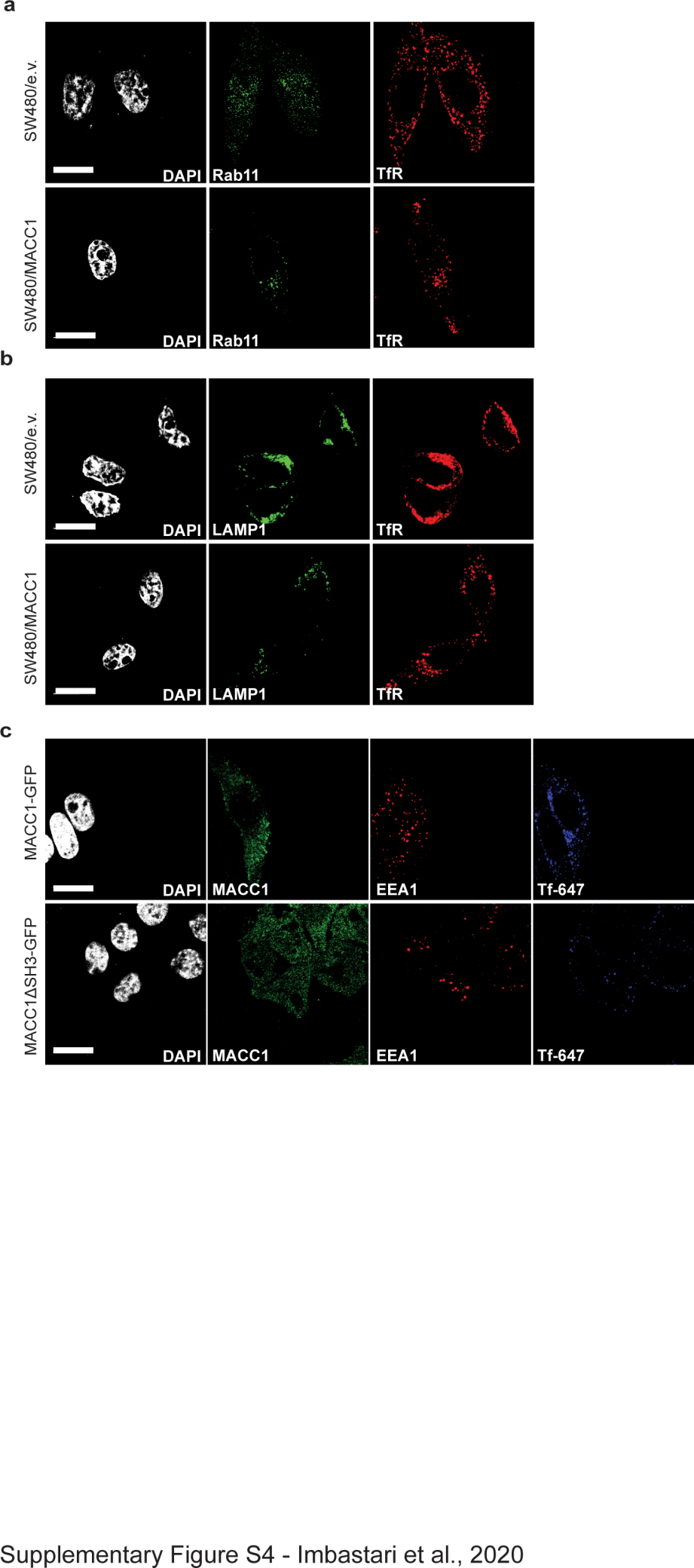


**Figure S4: Single channel images of the co-localization of TfR with Rab11, LAMP1 and EEA1, and triple staining of MACC1, EEA1 and Tf**

**(a-c)** Co-localization of TfR and Rab11 **(a)**, LAMP1 **(b)** and EEA1 **(c)** in SW480/e.v. and SW480/MACC1 cells after 15 min stimulation of TfR internalization with h‑Tf. Nuclei were stained with DAPI. Scale bar = 10 µm. **(d)** Co-localization of MACC1, EEA1 and Tf after triple-staining of SW480/MACC1‑GFP and SW480/MACC1ΔSH3‑GFP cells after 15 min stimulation of TfR internalization with Tf‑647. Nuclei were stained with DAPI. Scale bar = 10 µm.

Rab11 – Ras-related protein Rab11; TfR – transferrin receptor 1; LAMP1 - lysosomal associated membrane protein 1; EEA1 –early endosome antigen 1


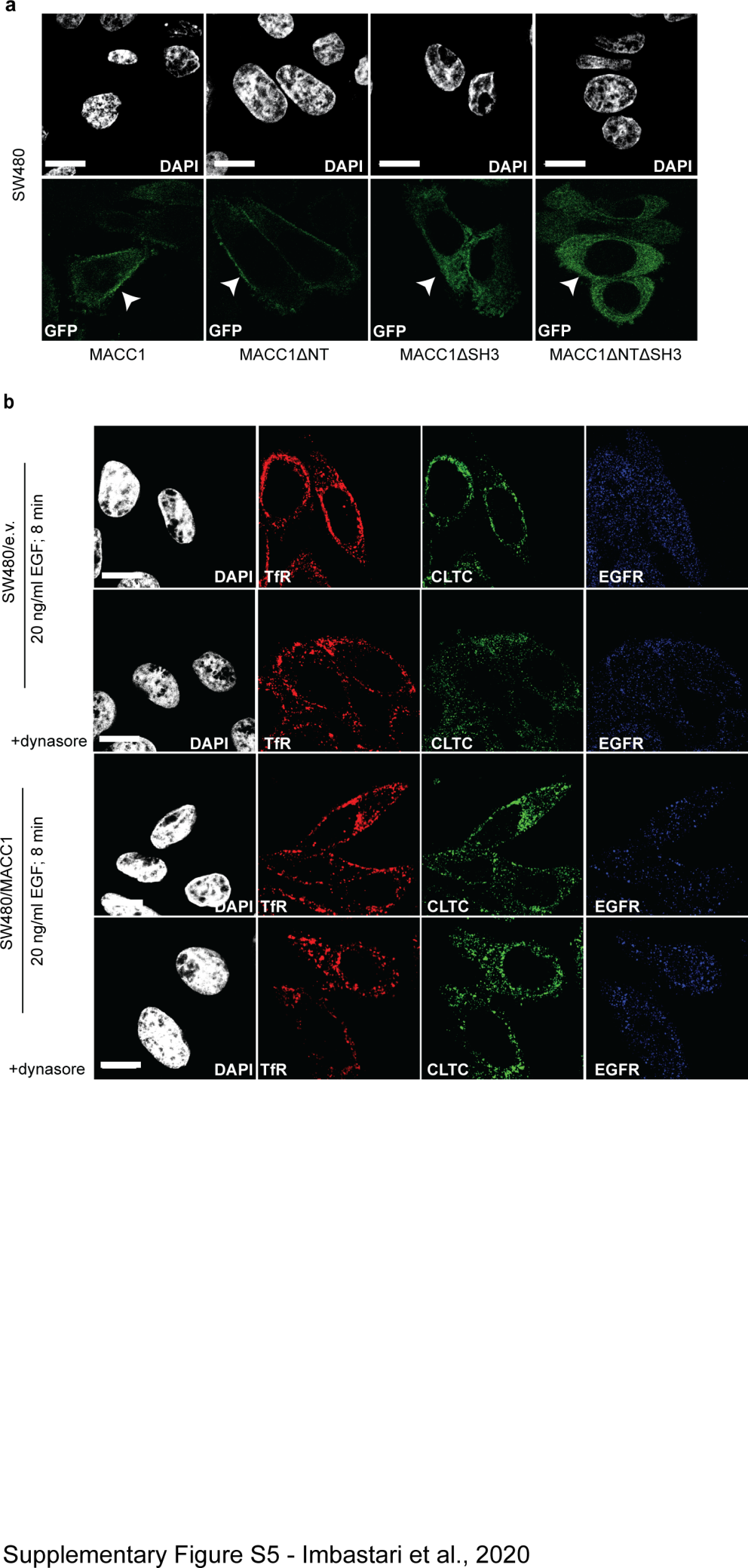


**Figure S5: Membrane localization of MACC1 deletion variants and single channel images of the co-localization of TfR with CTLC and EGFR**

**(a)** Cellular distribution of MACC1‑GFP and deletion variants by confocal fluorescence microscopy. Arrows indicate the plasma membrane. Nuclei were stained with DAPI. **(b)** Co-localization of TfR, CLTC and EGFR after triple-staining and 8 min treatment with 20 ng/ml EGF, in SW480/e.v. and SW480/MACC1 cells and in the presence or absence of the dynamin inhibitor dynasore. Nuclei were stained with DAPI. Scale bar = 10 µm.

NPF – interaction motif for Eps15-homology domains; DPF - interaction motif for AP‑2 complex; ZU5 – domain present in zona occludens 1 and uncoordinated‑5; UPA – domain found in uncoordinated‑5, p53-induced death domain protein 1 and ankyrins; SH3 – Src homology domain 3; DD – death domain; TfR – transferrin receptor 1; CLTC – clathrin heavy chain 1; EGF/R – epidermal growth factor/receptor.


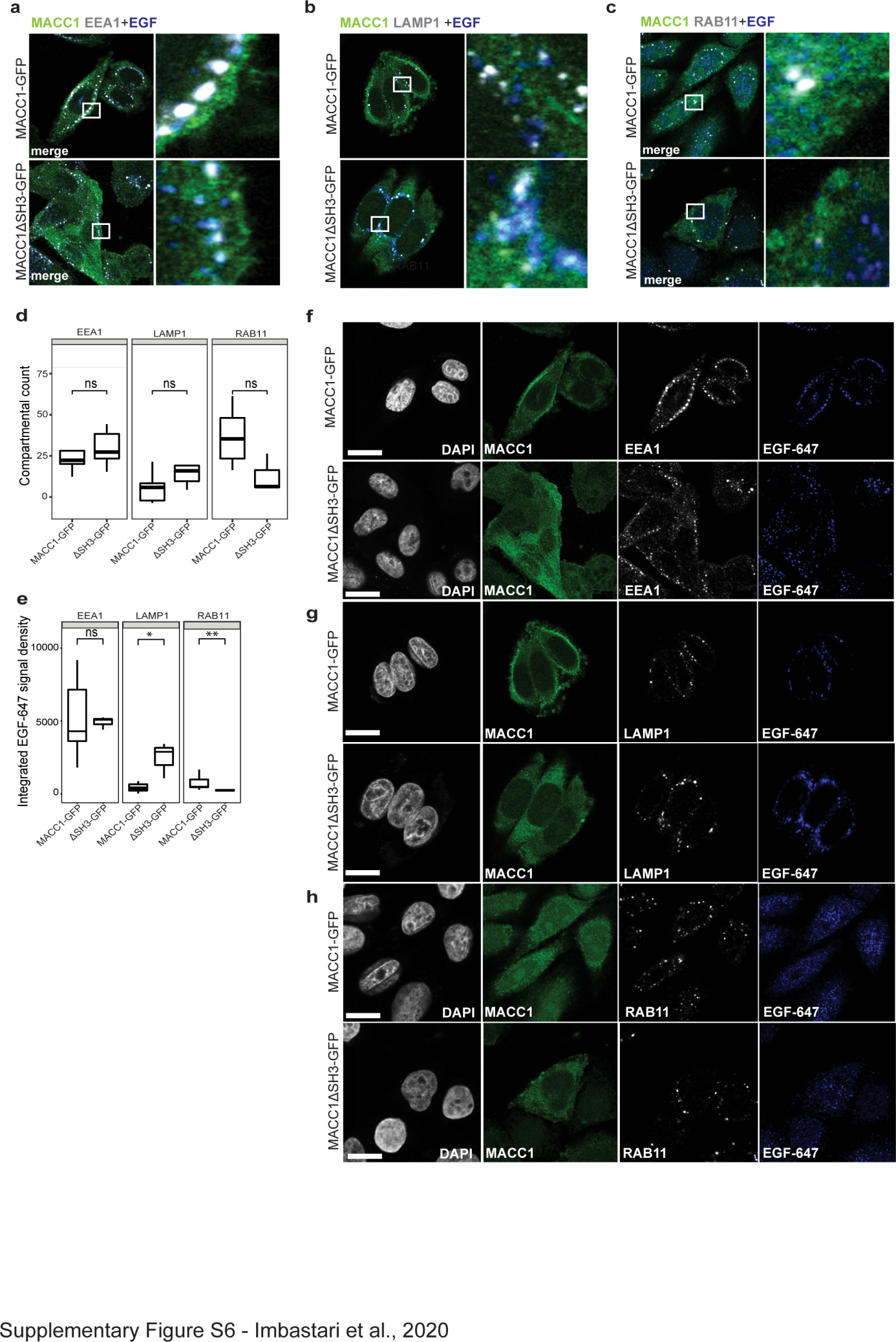


**Figure S6: Localization of EGF/EGFR protein complexes in dependency of functional MACC1**

**(a-c)** Co-localization of EGF-647 and EEA1 **(a)** LAMP1 **(b)** or RAB11 **(c)** compartments in SW480/MACC1-GFP and SW480/MACC1ΔSH3-GFP cells after EGF-647 stimulated EGFR internalization is shown. Scale bar = 10 µm. Indicated regions are displayed enlarged (10x). **(d)** Quantification of specific endosomal compartments, labeled with EEA1, RAB11, LAMP1 (respectively) in SW480/MACC1-GFP and SW480/MACC1ΔSH3-GFP cells after EGF 647-dependent EGFR internalization (n ≤ 10 cells; mean ± SEM). Statistical analysis was performed by Wilcox test. **(d)** Compartment-specific quantification of integrated EGF-647 signal density in EEA1-, LAMP1- and RAB11-marked compartments. Statistical analysis was performed by Wilcox test (n ≤ 10 cells; mean ± SEM, n=3). **(f-h)** Single channel images of the co-localization of MACC1, EGF-647 and EEA1 **(f)**, LAMP1 **(g)**, or RAB11 **(h)** in SW480/MACC1-GFP and SW480/MACC1ΔSH3-GFP cells after EGF 647-dependent EGFR internalization Nuclei were stained with DAPI. Scale bar = 10 µm. EGF/R – epidermal growth factor/receptor; EEA1 –early endosome antigen 1; LAMP1 - lysosomal associated membrane protein 1; RAB11 – Ras-related protein Rab11; * p < 0.05, ** p < 0.01


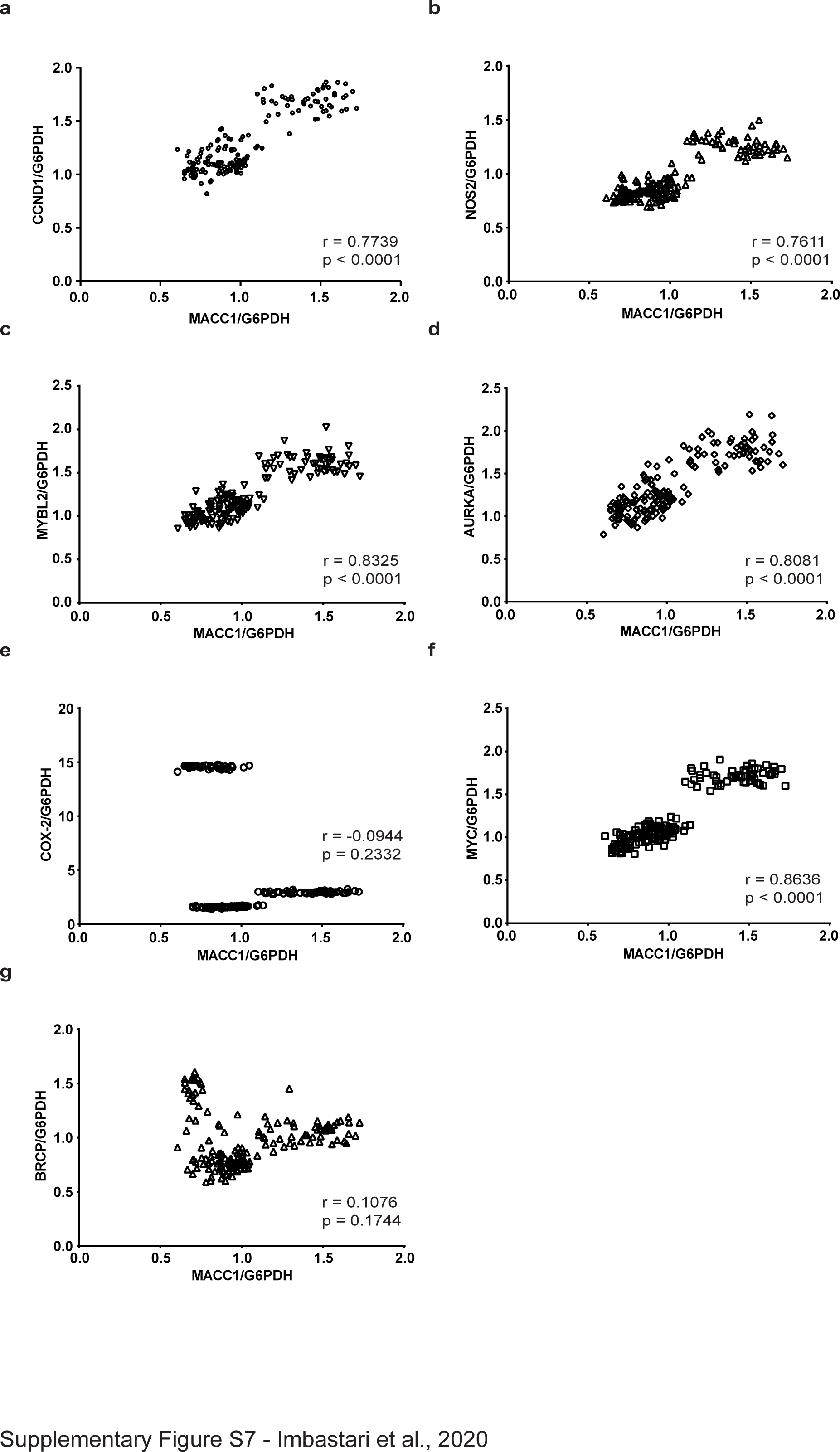


**Figure S7: Correlation of MACC1 and EGFR-target gene expression in microarray data of CRC patient tumors.**

**(a-g)** Publicly available sets of expression data of tumor tissue from four independent CRC patient cohorts were normalized to G6PDH and merged into a single file (n = 161). Correlation analysis of relative MACC1 expression (MACC1/G6PDH) and the expression level of EGFR-target genes validated a role of MACC1 in EGFR-dependent expression regulation in cancer cells.


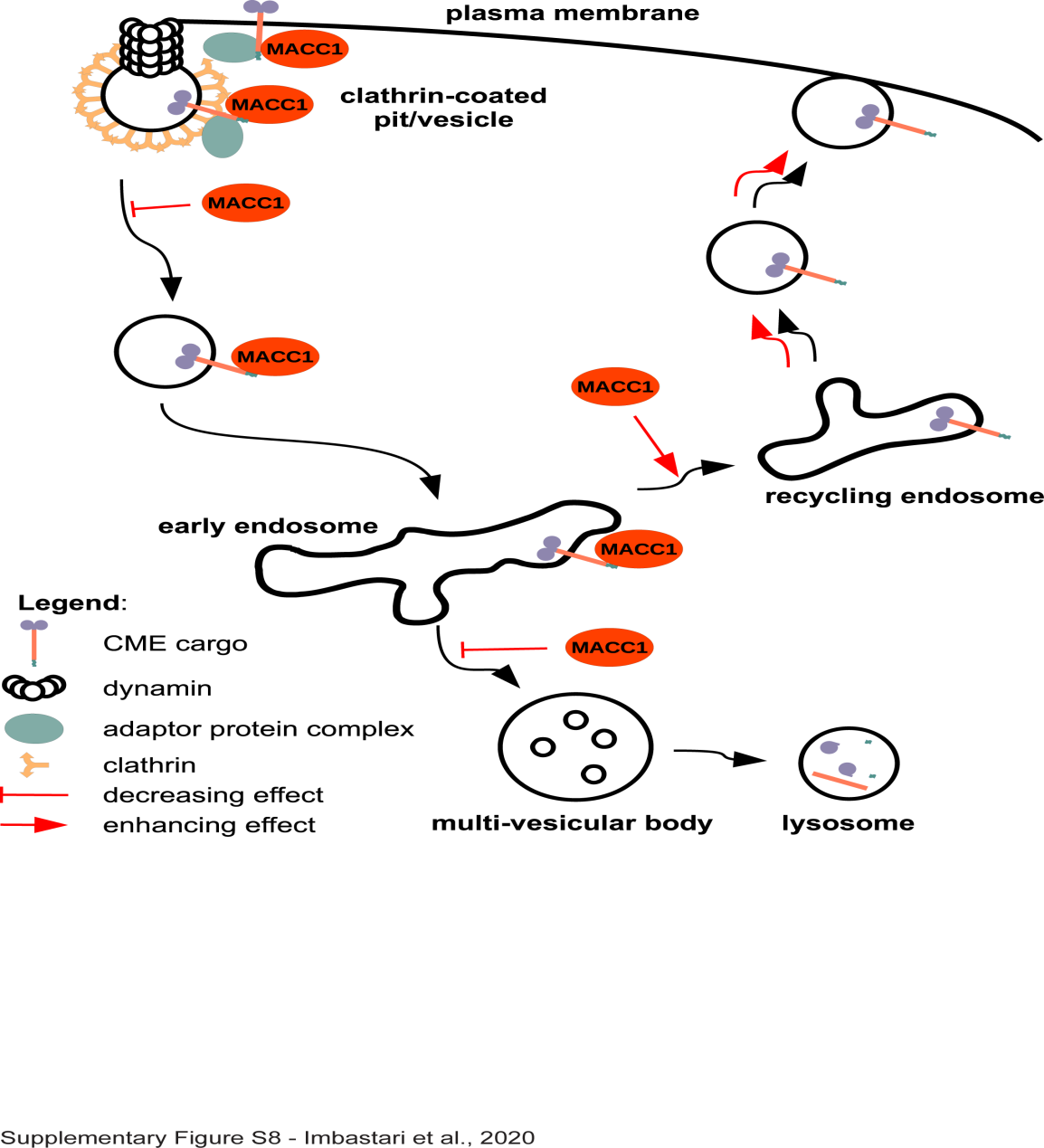


**Figure S8: Graphical summary: Schematic representation of the reported impact of MACC1 on CME and cargo fate.**

MACC1 interacts with cargo proteins, such as TfR or EGFR, as well as with accessory proteins for CME, including CLTC, DNM2 and AP‑2α. MACC1 stays associated with the cargo in CCVs and early endosomes. MACC1 overexpression decreases the uptake rate of cargo proteins and facilitates faster recycling, while decreasing its degradation. In case of EGFR, the enhanced recycling after CME at high MACC1 expression levels leads to faster and higher receptor phosphorylation and stronger and prolonged downstream signaling activity.

TfR – transferrin receptor 1; EGFR – epidermal growth factor receptor; CME – clathrin-mediated endocytosis; CLTC – clathrin heavy chain 1; DNM2 – dynamin‑2; AP‑2α – adaptor protein 2α; CCV – clathrin-coated vesicle
